# Vestibular-Evoked Cerebral Potentials

**DOI:** 10.3389/fneur.2021.674100

**Published:** 2021-09-21

**Authors:** Estelle Nakul, Fabrice Bartolomei, Christophe Lopez

**Affiliations:** ^1^Centre National de la Recherche Scientifique (CNRS), Laboratoire de Neurosciences Cognitives (LNC), FR3C, Aix Marseille Univ, Marseille, France; ^2^Institut de Neurosciences des Systèmes, Inserm, Aix Marseille Univ, Marseille, France; ^3^Service de Neurophysiologie Clinique, Hôpital Timone, Aix Marseille Univ, Marseille, France

**Keywords:** vestibular-evoked potentials, EEG, vestibular cortex, neuro-otology, vestibular system

## Abstract

The human vestibular cortex has mostly been approached using functional magnetic resonance imaging and positron emission tomography combined with artificial stimulation of the vestibular receptors or nerve. Few studies have used electroencephalography and benefited from its high temporal resolution to describe the spatiotemporal dynamics of vestibular information processing from the first milliseconds following vestibular stimulation. Evoked potentials (EPs) are largely used to describe neural processing of other sensory signals, but they remain poorly developed and standardized in vestibular neuroscience and neuro-otology. Yet, vestibular EPs of brainstem, cerebellar, and cortical origin have been reported as early as the 1960s. This review article summarizes and compares results from studies that have used a large range of vestibular stimulation, including natural vestibular stimulation on rotating chairs and motion platforms, as well as artificial vestibular stimulation (e.g., sounds, impulsive acceleration stimulation, galvanic stimulation). These studies identified vestibular EPs with short latency (<20 ms), middle latency (from 20 to 50 ms), and late latency (>50 ms). Analysis of the generators (source analysis) of these responses offers new insights into the neuroimaging of the vestibular system. Generators were consistently found in the parieto-insular and temporo-parietal junction—the core of the vestibular cortex—as well as in the prefrontal and frontal areas, superior parietal, and temporal areas. We discuss the relevance of vestibular EPs for basic research and clinical neuroscience and highlight their limitations.

## Introduction

The vestibular system has long been associated with postural, oculomotor, and autonomic reflexes. Recent studies from neuroscience and neurology have provided a large corpus of data showing that vestibular functions reach far beyond oculomotor and postural reflex control (1, 2). For example, vestibular signals have been involved in several aspects of spatial cognition and memory (3, 4), affective processing (5), personality (6), awareness (7), body representations (8), and self-consciousness (9).

The vestibular contributions to sensorimotor control, awareness, and cognition rely on neural pathways from the inner ear to the vestibular nuclei, thalamus, and cerebral cortex (10, 11), as well as on vestibular pathways to the cerebellum and basal ganglia (12). Functional magnetic resonance imaging (fMRI) or positron emission tomography (PET) studies combined with caloric and galvanic vestibular stimulation identified a large thalamo-cortical vestibular network in the human brain (11, 13–16). The vestibular cortex encompasses the parieto-insular and operculo-insular cortex, the MT/MST complex, inferior parietal lobe (angular and supramarginal gyri), somatosensory cortex, precuneus, frontal cortex (premotor cortex and frontal eye fields), cingulate gyrus, and the hippocampus. Although there seems to be no primary vestibular cortex, functional and anatomical data suggest that the parietal operculum (area OP2), the posterior insula, and/or the retroinsular cortex are the core area underpinning vestibular information processing (11, 15–18). The operculo-insular and retroinsular cortex is considered the human homologue of the parieto-insular vestibular cortex (PIVC) described in several non-human primate species (19, 20). Anatomical studies and direct electrophysiological recordings in non-human primates corroborate results from fMRI and PET studies regarding the localization of the vestibular cortex [reviewed in (10)].

Understanding vestibular projections to the central nervous system is crucial to foster the diagnosis of central vestibular disorders, which concern 25% of patients referred to otoneurology units specialized in dizziness and vertigo (21). We note that despite the progress made over the last 20 years to localize the human vestibular cortex, the spatiotemporal dynamics of vestibular information processing is still poorly described when compared to the wealth of data accumulated in non-human primates using single cell recordings [e.g., (22–24)]. This is mainly due to the limitations of the imaging techniques that were mostly used to identify the vestibular cortex (fMRI, PET). The long latency of hemodynamic response and poor sampling frequency of fMRI and PET did not allow to precisely describe the time course of vestibular responses in the human brain. Another limitation of fMRI and PET studies is that most of them did not use *natural vestibular stimulation*—with physiologically relevant patterns of angular and linear accelerations—as head movements are precluded in scanners [for exceptions, see (25), and more recently (26, 27)]. Instead, fMRI and PET studies have used *artificial vestibular stimulation*, including caloric, galvanic, acoustic, and magnetic stimulation of the vestibular receptors or nerve. Artificial vestibular stimulation do not allow to explore brain responses to the range of head translations and rotations involved in everyday activities (28), which may hamper a full understanding of the vestibulo-thalamo-cortical functions. Moreover, the use of artificial vestibular stimulation [such as caloric vestibular stimulation] in an MRI scanner may create conflict between vestibular signals—indicating self-motion—and visual, somatosensory, and interoceptive signals—indicating that the participant is motionless in the scanner. Thus, some of the brain areas shown to respond to vestibular stimulation in neuroimaging studies, such as the temporo-parietal cortex (29), may also be involved in monitoring, processing, or solving multisensory conflicts (30).

**Figure 1:**
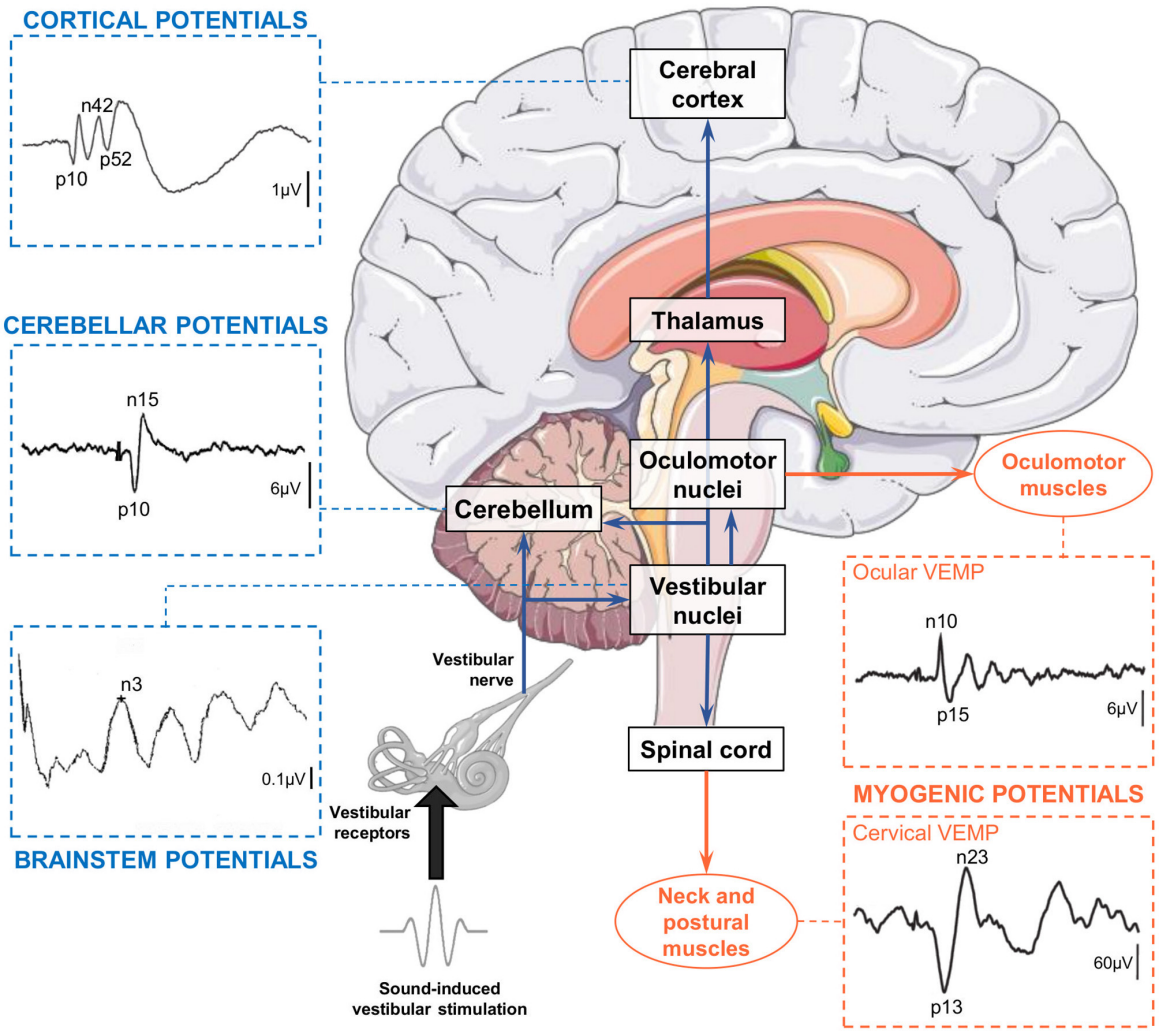
Electrical potentials evoked by sound-induced vestibular stimulation can be recorded along the pathways from the otolithic receptors to the central nervous system and muscles. Vestibular-evoked myogenic potentials (VEMPs) are recorded over extraocular, neck, and postural muscles. Cervical VEMPs reflect an inhibitory reflex and are recorded with electrodes over the sternocleidomastoid muscle ipsilateral to the stimulation. Ocular VEMPs reflect an excitatory reflex and are recorded with electrodes placed over the inferior oblique muscle contralateral to the stimulation. Both traces are adapted from (39). Vestibular-evoked cerebral potentials (VestEPs) are recorded from electrodes placed on the scalp or neck. Brainstem potentials are characterized by an n3 component observed under electrode P3 [this example recorded in a healthy participant is adapted from (40)]. Cerebellar potentials: Grand mean evoked potentials showing probable cerebellar components p10 and n15 observed under electrode P08 [illustration adapted from cf. Govender et al. (41) with permissions from Springer Nature]. Cortical potentials: Grand mean evoked potentials showing components p10, n42 and p52 observed under electrode FCz [illustration adapted from (42)]. Brain illustration from Servier Medical Art (smart.servier.com).

In contrast with fMRI and PET, electroencephalography (EEG) and evoked potentials (EPs) allow to study vestibular information processing with a resolution below the millisecond rather than seconds. Electroencephalography allows to detect, quantify, and analyze brain electrical activity, including responses to sensory stimuli (31). Importantly, EEG is compatible with natural vestibular stimulation (i.e., whole-body rotations and translations) that limits the induction of multisensory conflicts inherent to artificial vestibular stimulation. Rotatory chairs and whole-body motion platforms allow to explore a large range of vestibular stimuli with highly precise and reproducible motion parameters (32, 33). As EEG allows to measure brain responses within the first milliseconds after a sensory stimulation is applied, neurologists and neurophysiologists commonly use sensory EPs to assess the integrity and functioning of sensory systems. Both latency and source localization of somatosensory EPs (34), visual EPs (35) and auditory EPs (36, 37) are well-described and EP approaches are used worldwide in clinical routine. Regarding the vestibular system, the EP approach is well-developed to assess vestibulo-ocular and vestibulocollic reflex pathways (38) through electromyographic recordings above the oculomotor and neck muscles, respectively (Figure 1). Vestibular stimulation by air-conducted sounds and bone-conducted vibrations (43) are now commonly used to assess the latency and amplitude of *cervical* vestibular-evoked myogenic potentials (cVEMPs, recorded above neck muscles) and *ocular* vestibular-evoked myogenic potentials (oVEMPs, recorded above oculomotor muscles). However, we note that *cerebral* vestibular-EPs [referred to as Vestibular-Evoked Potentials (VestEPs) in line with (44) and (33)] recorded over the scalp using EEG or magnetoencephalography (MEG) have been explored since decades but are not yet part of the clinical vestibular assessment.

This article reviews findings from electrophysiological investigations of VestEPs in humans. We outline the advantages and limitations of different vestibular stimulation methods for EPs approaches. We then describe the spatiotemporal characteristics of VestEPs, distinguishing between those of probable brainstem, cerebellar, and cortical origins. Finally, we present potential applications of the VestEPs to otoneurology and to cognitive neuroscience.

## Vestibular Stimulation for Neuroimaging Studies and Their Application to Vestibular-Evoked Potentials

The scarcity in VestEPs studies is largely due to technical challenges to stimulate the vestibular system in a well-controlled and reproducible way. A variety of techniques has been used to activate the vestibular receptors or vestibular nerve in humans. These techniques fall into two main groups. One group involves *natural* vestibular stimulation using whole-body rotations or translations on motorized devices. These techniques are compatible with EEG recordings and EPs approaches, but they are to date not compatible with “online” fMRI and PET recordings. The other group of techniques involves *artificial* stimulation of the vestibular end organs in participants keeping their head fixed in space. Cold and warm CVS with air or water, binaural or monaural GVS and sound-induced vestibular stimulation (SVS) are the most common techniques. These stimulation techniques are fully compatible with neuroimaging and electrophysiological recordings. However, although they have been largely used in fMRI and PET studies, they have not often been used in EEG studies. This section briefly presents the main techniques for vestibular stimulation [for detailed reviews see (15, 45, 46)] with their advantages and limitations to measure VestEPs using EEG.

### Rotatory Chairs and Whole-Body Motion Platforms

Passive whole-body motion has been used to investigate VestEPs, mostly using rotatory chairs combined with EEG recordings (44, 47–54). To our knowledge, the first study presenting results from EEG recordings in participants sitting on a rotating chair was conducted by Greiner et al. (47). Chairs rotating around an earth-vertical axis stimulate the horizontal semicircular canals in participants sitting upright, and stimulate the vertical canals in participants lying supine or lying on their side (55). Rotations can also be applied only to the head, for example in lying participants with their head inserted and firmly held in a rotating drum (56). As summarized in a literature review by Ertl and Boegle (46), “most studies used smooth motion profiles like raised-cosine velocity profiles with peak velocities above 100°/s” or “used transient stimuli with duration shorter than 100 ms and peak accelerations up to 12,500°/s^2^” (32, 51, 57). Such controlled stimuli allow to study vestibular-evoked responses time-locked to different motion parameters (i.e., onset, offset, peak velocity). Voluntary, active head rotations with accelerations up to 12,000°/s^2^ have also been used (58, 59).

Linear motion platforms and tilting devices deliver natural stimulation to the otolithic receptors (the utricule and the saccule) (60). When compared to the processing of semicircular canal signals, there is only scarce description of how the vestibulo-thalamo-cortical system processes otolithic signals (61). Devices allowing whole-body translations are less common than rotating chairs in basic science laboratories and hospitals, which may have hampered the description of otolithic responses.

**Figure 2:**
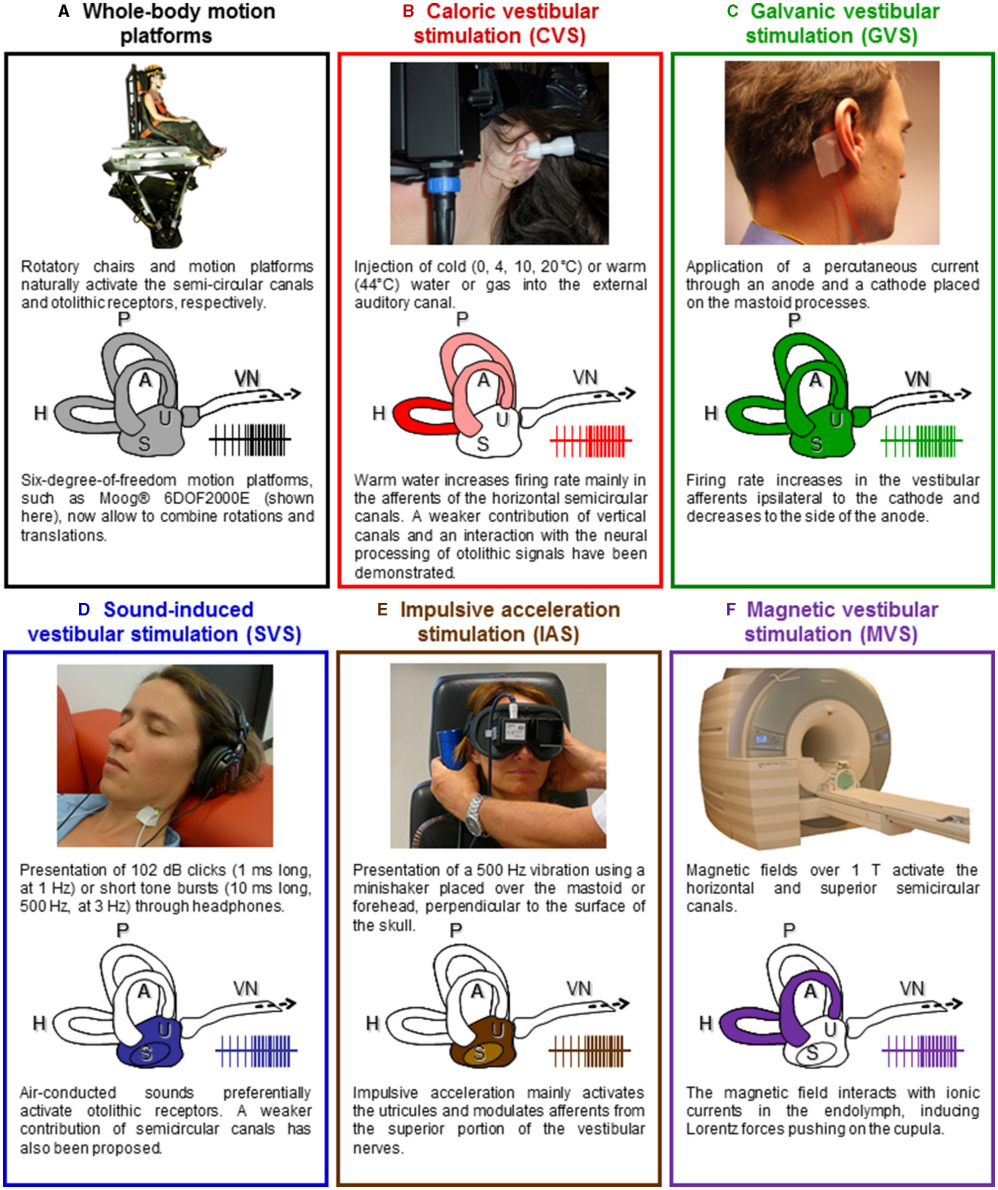
Vestibular stimulation techniques. **(A)** Rotating chairs stimulate the semicircular canals and linear motion platforms and tilting devices deliver natural stimulation to the otolithic receptors (the utricule and the saccule). New motion platforms with precise control of the amplitude, acceleration, and velocity of passively applied movements now allow to study responses to complex natural vestibular stimulation. Illustration adapted with permissions from (62). **(B)** Caloric vestibular stimulation (CVS) consists of irrigating the external auditory canal with warm or cold water or airflow. Caloric vestibular stimulation evokes a nystagmus and self-motion perception, often leading to vertigo and a sensation of dizziness. Illustration adapted from (15) with permissions from Elsevier. **(C)** Galvanic vestibular stimulation (GVS) consists of applying a weak transcutaneous current through an anode and a cathode placed over the mastoid processes. The cathode increases the firing rate in the ipsilateral vestibular afferents, while the anode decreases it. GVS stimulates simultaneously all otoliths and semicircular canals afferents. Illustration adapted from (15) with permissions from Elsevier. **(D)** Auditory stimuli such as clicks and short-tone bursts can stimulate the otolithic receptors. Illustration adapted from (15) with permissions from Elsevier. **(E)** 500 Hz vibrations applied on the mastoids or the forehead using a minishaker stimulate the otolithic receptors and induce ocular and cervical VEMPs. Illustration adapted from (63). **(F)** Based on the analysis of the nystagmus it evokes, magnetic vestibular stimulation (MVS) is thought to activate the horizontal and superior semicircular canals. It offers a way to produce long-duration vestibular stimulation, equivalent to a constant angular acceleration on a motion platform. A, H, P, anterior, horizontal, and posterior semicircular canals; S, saccule; U, utricule; VN, vestibular nerve.

New motion platforms with precise control of the amplitude, acceleration, and velocity of passively applied movements now allow to study responses to complex natural vestibular stimulation. Six-degree-of-freedom motion platforms, such as the Moog® 6DOF2000E (Figure 2A), provide comparisons with studies in macaques that have used the same platform to record single cell responses to whole-body rotations and translations (24, 64).

Rotatory chairs and whole-body motion platforms are incompatible with fMRI and PET, because head movements are precluded in scanners. To circumvent this issue, *passive* whole-body rotations and translations followed by offline PET recordings have been used in a recent study (65). Although this study is original in that it reports predominant bilateral activation in the deep part of the Heschl's gyrus, overlapping with the posterior insula, in response to natural whole-body motion, the response was recorded offline, and does not reflect the spatiotemporal pattern of vestibular information processing. Few studies have used blood-oxygen-level-dependent (BOLD) recordings after voluntary *active* head rotations to investigate vestibular responses (25–27). A main limitation of this approach is that vestibular responses are reduced in the brainstem and cerebellum during active compared to passive movements, as shown in animal studies (66–68). In addition, intraparietal neurons respond to different directions of movement depending on whether the movement is active or passive (69, 70). These differences in vestibular processing limit a direct comparison of neuroimaging data using active head motion with studies using passive body motion.

#### Application to VestEPs

Electroencephalography remains the best and most direct method to analyze the spatiotemporal dynamics of brain responses to natural vestibular stimuli, given its high temporal resolution below the milliseconds and its compatibility with recordings during natural, passive whole-body motion. One must, however, consider that rotatory chairs and motion platforms for body translations can induce mechanical and electromagnetic artifacts in the EEG signal, besides artifacts due to reflexive eye movements (i.e., vestibulo-ocular reflex) and muscles contractions (i.e., vestibulocollic and vestibulospinal reflexes). Rotatory chairs and motion platforms also generate auditory noise that needs to be controlled for. Finally, it should be noted that body rotations and translations activate the somatosensory and interoceptive systems, respectively, due to the pressure of the body against the chair or to the movement of bodily fluids, which can hardly be diminished.

### Caloric Vestibular Stimulation

Caloric vestibular stimulation (CVS) is the most common technique to evaluate the semicircular canals functions (see Figure 2B). It consists in applying warm (≥44°C) or cold ( ≤ 30°C) water (or gas) in the auditory canal of participants lying supine, with their head tilted 30° forward. The fluid creates a temperature gradient in the semicircular canals, which induces an endolymphatic flow activating the hair cells in the crista ampullaris. The firing rate in the vestibular afferents increases or decreases accordingly to the increase or decrease in temperature in the inner ear. Caloric vestibular stimulation mostly activates the horizontal semicircular canal, with a weaker contribution of the anterior and posterior canals (71). This stimulation induces a nystagmus toward the stimulated ear with hot water or gas and induces a nystagmus toward the opposite ear with cold water or gas. These oculomotor responses are accompanied by complex sensations of rotation, floating, and tilting. These manifestations occur only after several seconds of stimulation and a clear onset is often difficult to determine. They can also last several minutes after the end of the stimulation. Caloric vestibular stimulation is fully compatible with fMRI, PET, EEG, and MEG and has been used in the pioneer vestibular neuroimaging studies about 40 years ago (72, 73). Recent neuroimaging studies showed that CVS activates several cortical areas, such as the inferior parietal lobule, superior temporal gyrus, insula, frontal cortex, and frontal eye fields as well as hippocampal, parahippocampal, and thalamic regions [(74–80); for a detailed review see (15)].

#### Application to VestEPs

Caloric vestibular stimulation has been used in early studies of VestEPs, especially in epileptic patients (81–85). These studies showed that CVS modulates brain rhythms (e.g., alpha rhythm desynchronization) and can trigger seizures in predisposed patients. However, CVS does not seem appropriate for EPs approaches for several reasons. First, the nystagmus evoked by CVS can create important artifacts to the EEG recordings. Second, as the exact onset of the effects of caloric stimulation on vestibular receptors is difficult to determine, this precludes EP approaches. Third, CVS cannot be repeated many times in a short period of time, which is required to calculate EPs. Finally, CVS activates the somatosensory, thermoceptive and nociceptive sensory systems, leading to unspecific activations of extravestibular pathways.

### Galvanic Vestibular Stimulation

In contrast to CVS, galvanic vestibular stimulation (GVS) activates the vestibular end organs with a temporal precision under the microsecond. Galvanic vestibular stimulation consists in the application of a small transcutaneous electrical current (in general up to 5 mA) through a cathode and an anode placed on the skin over the mastoid processes (see Figure 2C). Galvanic vestibular stimulation can be applied monaurally (electrodes are on the same ear) or binaurally (electrodes are placed on the opposite ears), with continuous electrical stimulation, single square-wave pulse, or trains of pulses. Galvanic vestibular stimulation is thought to directly modulate the firing rate of the vestibular afferents (86), although a GVS may also stimulate the vestibular hair cells (87). The cathode increases the firing rate in the vestibular afferents, while the anode decreases it [reviewed in (88)]. Galvanic vestibular stimulation is an artificial vestibular stimulation in that it bypasses the mechanoelectrical transduction in the hair cells and activates afferent fibers from receptors that would never be activated together during naturalistic head movements. Continuous GVS induces, almost instantaneously, complex sensations of combined translation and rotation, which orientation and intensity can be modulated by the direction and intensity of the applied current. As early as the 1990s, neuroimaging studies have used GVS to localize the vestibular cortex. They identified areas such as the supramarginal gyrus, precuneus, posterior cingulum, superior and middle temporal gyrus, insula, frontal areas and frontal eye fields, inferior and superior occipital gyrus as well as hippocampal, parahippocampal, and thalamic areas [(89–93); for a detailed review see (15)].

#### Application to VestEPs

Trains of short electrical pulses, such as those used to evoke cVEMPs [e.g., 2-ms pulses at 5 Hz; (94)], generally do not evoke self-motion perception and are theoretically ideal to measure VestEPs. However, GVS can evoke muscular responses such as cVEMPs (94) also time-locked to the stimulation, which can contaminate VestEPs recordings using EEG. Galvanic vestibular stimulation is a transcutaneous stimulation that activates the somatosensory—and sometimes the nociceptive—system. More importantly, GVS generates electromagnetic artifacts that affect EEG recordings and may not be suppressed, preventing the observation of short latency VestEPs. One early study combined continuous GVS with EEG to investigate long latency VestEPs and described a series of positive and negative components with an onset latency around 60–80 ms which could last up to 500 ms after the stimulation (95). To our knowledge, only one EEG study has recently identified VestEPs of middle and long latency evoked by 3 ms square-wave pulses (96).

### Sound-Induced Vestibular Stimulation

Sound-induced vestibular stimulation (SVS) offers the precise timing of GVS without electromagnetic artifacts and seems therefore particularly appropriate for event-related EEG studies (see Figure 2D). Short sounds are highly reproducible and repeatable stimuli whose onset and offset can be controlled with a millisecond precision. Short high sound pressure clicks at intensities around 100 dB-SPL and short tone-bursts at 500 Hz pressurize and activate otolithic receptors (43, 97, 98). Sound-induced vestibular stimulation is widely used in otoneurology to compare the latency and amplitude of cVEMPs and oVEMPs after stimulation of the right and left ear separately (38, 99, 100). Sound-induced vestibular stimulation does not seem to induce any vestibular perception although this has never been thoroughly investigated. Sound-induced vestibular stimulation has been used in neuroimaging studies of the vestibular system and revealed otolithic projections to frontal, parietal, and cingulate regions, similar to areas revealed using semicircular canal stimulation (101–103).

#### Application to VestEPs

Sound-induced vestibular stimulation allowed to identify VestEPs and to describe components of short (40, 104–109), middle (42, 108, 110, 111), and late latency (42, 110, 112). As described below, short, middle, and late VestEPs evoked by SVS have been associated to different generators along the vestibulo-thalamo-cortical pathways, so that SVS likely allows to study the spatiotemporal processing of vestibular information from the periphery to the cortex.

Vestibular-evoked potentials and auditory EPs show similar latencies and some responses to SVS appear to contain both auditory and vestibular contributions. Different techniques can then be used to disentangle them. As SVS de facto activates the auditory system, studies have used control auditory stimuli, modulating either the intensity or the frequency of sounds to separate vestibular and auditory responses (108, 111, 113). Most studies of VestEPs using SVS have used sounds below and above the vestibular threshold, determined as the intensity above which sounds evoke VEMPs for a given ear in a given individual (42, 107, 108, 110, 113–115). This allows to identify VestEPs, which only appear for SVS above the vestibular threshold, from auditory components appearing for SVS below and above the vestibular threshold. The validity of this approach has been confirmed in recent fMRI studies. An independent component analysis revealed a specific increase in BOLD response for SVS above the vestibular threshold in areas such as the insula, precuneus, inferior parietal lobule, middle cingulate cortex, and cerebellar uvula (116). Subsequent parametric analyses revealed vestibular-auditory integration in the caudal part of the superior temporal gyrus and posterior insula (117). However, some authors argued that in EEG studies it might be difficult to disambiguate between auditory and vestibular components because the time course of VestEPs during whole-body translation and the time course of auditory EPs overlap (33). In conclusion, SVS seems to be a useful and convenient technique to evoke VestEPs and study their cerebral origin using source analysis, provided that relevant controls and analyses are used to disentangle them from auditory components.

### Impulsive Acceleration Stimulation

Impulsive acceleration stimulation [IAS; (46)] also referred to as “bone-conducted” stimulation (41), can be applied using a minishaker placed over one of the mastoids or on the forehead, at the hairline (Fz), perpendicular to the skull surface (see Figure 2E). Five hundred Hz vibrations stimulate the otolithic receptors and induce ocular and cervical VEMPs (43, 118, 119). These can be used to investigate unilateral vestibular loss, for example by comparing responses below both eyes (63).

#### Application to VestEPs

Impulsive acceleration stimulation has been used in a few studies to evoke VestEPs, as it creates rapid and highly reproducible translational accelerations up to 0.2 g (108, 120). However, IAS activates the somatosensory and auditory systems and can cause small head movements creating artifacts in fMRI and EEG studies. For these reasons, IAS remains rarely used for the study of central vestibular projections.

### Magnetic Vestibular Stimulation

Magnetic vestibular stimulation (MVS) recently emerged as a new method to stimulate vestibular receptors (see Figure 2F). Magnetic fields over 1 T can induce a nystagmus in healthy participants that is absent in patients with a bilateral vestibular failure (121–123). Based on the analysis of the nystagmus it evokes, MVS is thought to activate the horizontal and superior semicircular canals (124). Magnetic vestibular stimulation interacts with ionic currents in the endolymph, inducing Lorentz forces pushing on the cupula. It offers a way to produce long-duration vestibular stimulation, equivalent to a constant angular acceleration on a rotatory chair. Accordingly, MVS over 3 T can induce sensations of rotation. Magnetic vestibular stimulation has been shown to modulate the BOLD response in vestibular and oculomotor areas, including the anterior cingulum, cerebellar vermis, and calcarine sulcus (125). Magnetic vestibular stimulation can be used as vestibular stimulation in conjunction with resting-state fMRI or fMRI studies of cognitive processes.

#### Application to VestEPs

Electrophysiological recordings can now be arranged in an MRI bore and several studies showed that EEG, with event-related potential approaches, can be recorded simultaneously as fMRI (126–128). However, MVS has several major caveats for EP approaches, which have been reviewed in Ertl and Boegle (46). Mostly, MVS precludes the application of repeated stimuli with a clear onset: as the magnetic field of the scanner is constant, MVS does not allow to compare changes in brain activity due to MVS with respect to a baseline (without MVS) with an event-related potential approach. Magnetic vestibular stimulation also induces a nystagmus, that needs to be controlled for or inhibited to avoid muscular artifacts in the EEG signals. Of note, the magnetic field interferes with electrophysiological recordings and careful artifact removal is required [e.g., (129, 130)].

### Intraoperative Vestibular Nerve Stimulation

Direct electrical stimulation of the vestibular nerve can be combined with EEG recordings in patients undergoing vestibulocochlear nerve surgery. This was performed in rare studies during unilateral vestibular neurectomy for intractable Menière's disease and during neuroma resection (131–133).

#### Application to VestEPs

Intraoperative vestibular nerve stimulation is close to early electrophysiological investigations of the vestibular cortex in cats and monkeys (134, 135) or to recent studies in rodents (136), which applied electrical stimulation to the vestibular nerve. Early human studies, using montages with only few electrodes, did not provide information regarding the generators of the VestEPs (131, 132). Electrical stimulation may spread to the facial nerve or the acoustic nerve and general anesthesia may alter vestibular information processing.

### Conclusion

Major shortcomings have been emphasized for natural and artificial vestibular stimulations when neuroimaging the vestibular system is concerned (15, 46). To date, natural vestibular stimulation is not compatible with neuroimaging techniques with high spatial resolution such as fMRI. Most neuroimaging studies so far have used artificial stimulation to study the vestibular system with a high spatial resolution but a poor temporal resolution. By contrast, EEG has a high temporal resolution and is compatible with both natural and some artificial vestibular stimulations. However, EEG is known for its relatively low spatial resolution and the difficulty to accurately identify subcortical generators of signals recorded on the scalp. In addition to these issues of compatibility between stimulation and recording techniques, most artificial vestibular stimulation (and natural vestibular stimulation to a lesser extent) co-activate other sensory receptors. This includes mostly activation of auditory, somatosensory, interoceptive, as well as sometimes nociceptive systems, which are difficult to control for in neuroimaging studies. Moreover, artificial vestibular stimulation can induce sensory conflicts between vestibular information and other senses, contrary to natural vestibular stimulation.

With these limitations in mind, it seems feasible to investigate the spatiotemporal dynamics of vestibular information processing in the human central nervous system by carefully adapting the vestibular stimulation to each recording technique and using the necessary control conditions. The next section focuses on how VestEPs help understand the spatiotemporal dynamics of vestibular information processing.

## Vestibular-Evoked Potentials

Pioneering work described VestEPs in humans as early as the 1960s (47, 137, 138). Interestingly, VestEPs were also described in several animal species during the 1960s or the following decade using similar approaches [see for example studies in guinea pigs: (139); cats: (48, 140); non-human primates: (141)]. Early research focused on the influence of rotatory vestibular stimulation on EEG signals in epileptic patients. These studies showed that vestibular stimulation could activate temporal epileptic foci and sometimes trigger seizures (47, 85, 142–144). Another line of studies compared scalp responses between healthy participants and patients with a bilateral vestibulopathy or between the two sides in patients with a unilateral vestibular loss. They confirmed the existence of a vestibular response under scalp electrodes. The most consistent finding was a suppression of alpha rhythm over the temporo-parietal junction (47, 82, 143). Finally, early studies using CVS reported similar effects on EEG signals, showing that cerebral responses could also be evoked by artificial vestibular stimulation (82–85).

On the grounds of these pioneering studies and after improvement in recording techniques, VestEPs were described more precisely. Supplementary Table 1 summarizes the studies considered in this review, together with the latencies and scalp location of the main VestEPs, as well as the electrode montages used to record them. To facilitate the literature review, we have classified VestEPs as short (<20 ms), middle (20–50 ms), and late (> 50 ms) depending on their peak latencies and their most probable generators, similarly to the classification of auditory EPs (145). In the text and in the figures, we chose to homogenize the report of the VestEPs components by indicating their positive (p) or negative (n) polarity followed by their reported peak latency (or average latency) expressed in ms post-stimulation onset. We therefore avoid the use of general labels relative to the order of appearance of the components, such as P1, N1, P2, and N2, which refer to very different latencies in different studies using different paradigms and stimulation parameters. The purpose of this is not to redefine common component names but to help the reader compare components latency and polarity in a simple way and avoid confusion.

## Short Latency Vestibular-Evoked Potentials

Vestibular-evoked potentials with a latency below 10 ms have been related to signal conduction in the vestibular nerve and vestibular information processing in the vestibular nuclei (40, 56, 57, 104–106, 109, 132, 133, 146, 147). Vestibular-evoked potentials with a peak latency between 10 and 20 ms have been attributed to myogenic, cerebellar or cortical sources (41, 42, 56, 57, 107, 108, 110, 113–115, 120, 133, 148–150). In this section, we describe the short latency components that emerge across the studies and briefly discuss their origin.

### Short Latency Responses Under 10 ms

Responses with the shortest latency have been observed during perioperative stimulation of the vestibular nerve. An early study using direct electrical vestibular nerve stimulation in nine patients operated on for intractable Menière's disease revealed a negative potential with a latency of 2 ms and an amplitude of 0.5 μV (132). This response was suppressed after vestibular neurectomy, indicating its vestibular origin. In a later study, a similar stimulation elicited VestEPs with an onset of 3.5–5 ms and peak latency of 9.5 ms in 11 patients with 30 electrodes on the scalp (133). These responses were recorded bilaterally, but the larger responses were observed under ipsilateral electrodes at the lower part of the temporal scalp region.

A series of studies using rapid and passive head rotations in the yaw plane, consisting of impulses at 10,000°/s^2^, report similar responses. Some studies identified a forehead positive peak at 3.5 ms, a negative peak at 6 ms, and a positive peak at 8.4 ms (56, 57). Another study recorded a response onset around 2.2 ms, followed by a positive peak at 2.9 ms, and other components with peak latencies at 5.1, 7.0, and 8.6 ms (147). The responses amplitude ranged from 0.3 to 0.6 μV. Horizontal lateral translations also triggered responses with 3 and 6 ms peak latencies (146). Impulsive acceleration stimulation also evoked components with latencies of 1.9, 2.4, and 4.5 ms (151). Of note, skin surface recordings and intracranial recordings in the cat vestibular nuclei with the same acceleration impulses revealed that irregular neurons responded with a 3.5 ms latency to the onset of the head acceleration (152, 153). The authors proposed that the component with an onset around 2 ms reflects vestibular nerve activity, whereas the following components are of vestibular nuclei origin (152, 153).

More recent studies looking for vestibular components in the brainstem auditory-evoked potentials (BAEPs) have confirmed and extended such results. Brainstem auditory-evoked potentials are a standard for the clinical evaluation of hearing and brainstem auditory pathways (37). They consist of five to six vertex positive waves and likely present vestibular components of saccular origin. This was first suggested by studies in guinea pigs showing preserved short latency far field components after cochlear hair cells destruction (154, 155). In humans, a similar 3 ms latency negative peak, referred to as the n3, was identified using a classical BAEPs montage with loud clicks (40, 104, 109). Air-conducted tones pips, which delay BAEPs, induced a response similar to the n3, but with a 5 ms latency, referred to as the n5 (105, 106). The n3 and n5 are likely of vestibular origin, as they are found in deaf patients (104), but are absent in vestibular-defective patients, even when they show preserved hearing and a residual caloric nystagmus (109).

The n3 can be recorded at the vertex and the n5 is best observed ipsilaterally to the acoustic stimulation, over parietal areas. The short latency of the n3 and n5 components suggest that they are far-field potentials originating from the vestibular nuclei (104). Their absence in multiple sclerosis patients with demyelination in the lower pons confirms this origin (40, 105). In addition, an n6 component, also evoked by SVS but independent of the n5, was maximally recorded over the parieto-occipital area (106). Because it appears approximately 1 ms after the n5, this component has been proposed to originate in the rostral pons or the midbrain (106).

### Short Latency Responses Above 10 ms

Vestibular-evoked potentials between 10 and 20 ms are the most investigated, and several components have been identified. These components are often considered as part of a biphasic wave (such as the p10–n15 and n15–p21 waves) or have been described as individual components with a given peak latency (such as the p10 and p21). We summarize below the main individual components reported in the literature (Supplementary Table 1).

#### Component p10

Several studies have described a positive component with an onset latency of 6–7 ms and a peak latency around 10 ms after whole-body rotations (56, 57), SVS (42, 107, 108, 110, 114, 115, 149), and direct electrical nerve stimulation (133). Several VestEPs seem to appear at this latency because they are recorded under different electrode locations. Using SVS, it was possible to show that the p10 (as well as subsequent components: n15, n17, and p21; see below) was of vestibular origin. The p10 (as well as n15, n17, and p21) was present in a patient with hearing loss and preserved vestibular function, but was abolished in a patient with impaired vestibular function and preserved hearing (107). In addition, it was absent for SVS below the vestibular threshold (i.e., the intensity at which VEMPs appear) and was observed above this threshold (42, 107, 108, 110).

**Figure 3:**
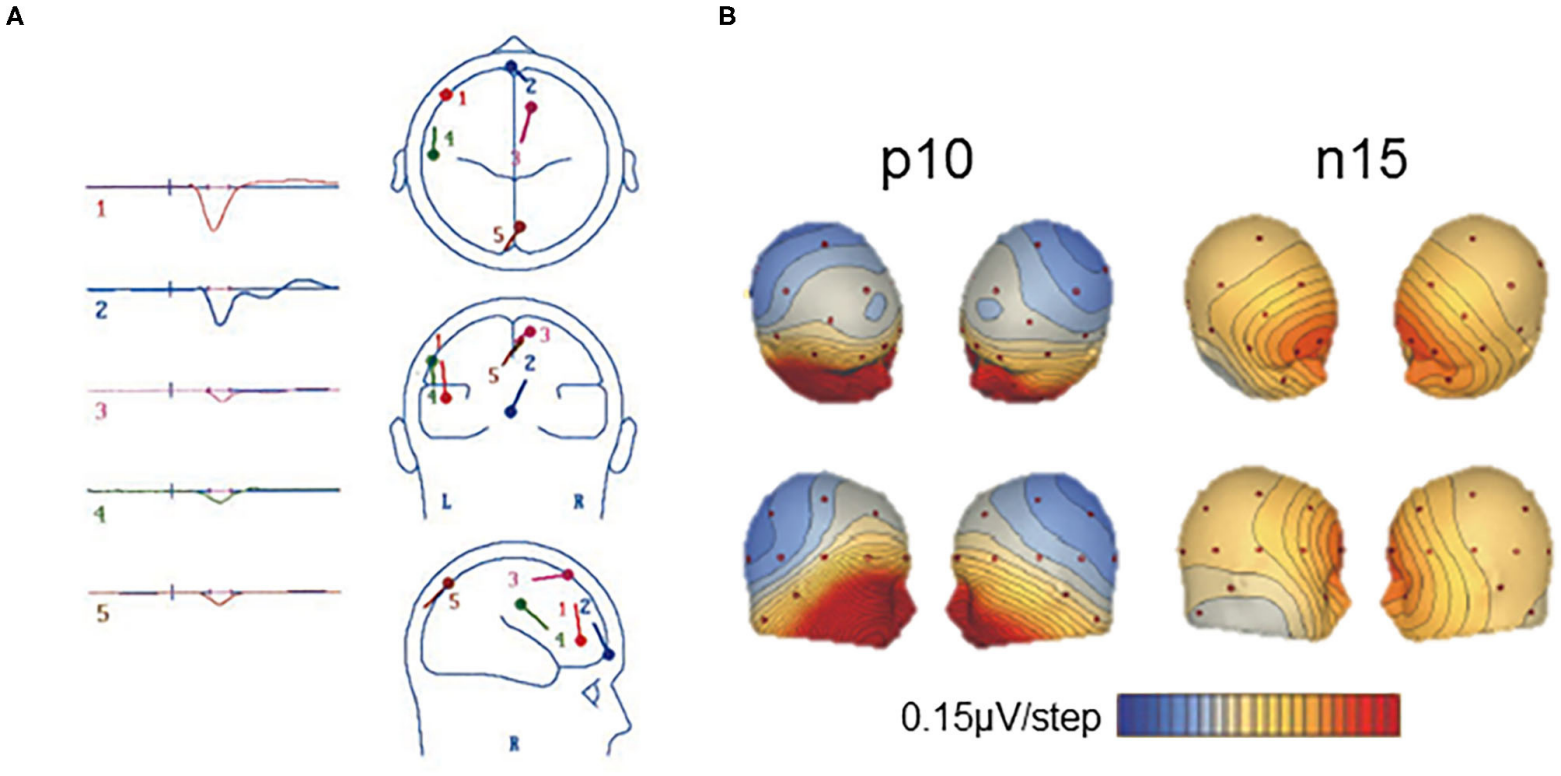
Short latency vestibular-evoked cerebral potentials (VestEPs). **(A)** Source dipole analysis of grand average VestEPs with latencies under 10 ms. VestEPs were triggered by electrical stimulation of the vestibular nerve during surgery. Five dipoles were identified and localized: dipole 1 (red) is at the limit of the ipsilateral frontal and prefrontal lobes, dipole 2 (blue) is on the transverse frontopolar and/or frontomarginal gyrus of the prefrontal lobe, close to the midline, dipole 3 (pink) is on the contralateral anterior portion of the supplementary motor area (around the supplementary eye fields); dipole 4 (green) is on the ipsilateral temporoparietal area; dipole 5 (brown) is on the contralateral superior occipital gyrus, although close to the midline. Adapted from (133) with permissions from Springer Nature. **(B)** Voltage maps of the p10 and n15 evoked by left sound-induced vestibular stimulation. Positive potentials are indicated in blue and negative in red and contours are spaced at 0.15 μV. Adapted from (108) with permissions from Elsevier.

Vestibular-evoked potentials with a latency near 10 ms were first showed frontally (56, 57, 133) or maximal at central electrode Cz with a small ipsilateral lateralization (107). De Waele et al. (133) proposed that such VestEPs reflect activation of several cortical areas. A dipole source analysis showed, within 6 ms, parallel activation of the ipsilateral temporo-parietal cortex, prefrontal, and/or frontal lobe, supplementary motor area, and contralateral parietal cortex [(133); Figure 3A]. These findings are in line with the observation that the posterior part of the postcentral gyrus (area 2) is activated within 5–6 ms after electrical vestibular nerve stimulation in the rhesus monkey (135).

A p10 component, followed by an n17 component, has also been observed at parietal electrode Pz [(108); Figure 3B] or at the inion (115). Several generators have been proposed for the p10 and n17 components. As the p10/n17 is concomitant to the oVEMP biphasic wave n10/p17, a myogenic or cerebral generator has been proposed (108, 113). Subsequent studies found that the p10 mirrors a frontal or infra-ocular n10 response (110) and supported the idea that they are two distinct responses (115).

A line of research suggests that the p10 may originate from the cerebellum (108, 110, 115, 149). Sound-induced vestibular stimulation evoked a p10/n17 response at occipital electrodes (PO7 and PO8) contralateral to the stimulated ear and at the inion (Iz), together with a n10/p17 complex under electrodes placed over the splenius muscles to record cerebellar activity (115). A source analysis found the contralateral cerebellum as the most likely origin of these responses (115). The fact that, as for the oVEMP n10/p17, the p10/n17 depends on gaze direction (115) indicates either a myogenic origin or the recording of cerebellar or cerebral mechanisms to regulate ocular responses to vestibular stimulation. To further investigate the cerebellar origin of these VestEPs, recent studies used extended EEG montages to record the electrocerebellogram, with electrodes over, laterally to and below the posterior fossa, thus over the inferior cerebellum (41, 120, 150, 156). With such montages, IAS revealed a p12/n17 biphasic wave (41, 120, 150). A source analysis showed the cerebellar origin of the p12/n17 (150). It was argued that the response could not be myogenic because neck muscles were relaxed during recordings, the response was lateralized, and the waveforms differed from those of cVEMPs (41). Although these recent results are very promising, one cannot exclude that neck muscle relaxation does not fully abolish a potential muscular contribution to the observed responses.

The above-mentioned results are not only encouraging for the electrophysiological investigation of the spatiotemporal dynamics of vestibular information processing, but also for the non-invasive electrophysiological study of cerebellar functions in general. The study of the cerebellum using EEG is indeed controversial due to the structure of the cerebellum, traditionally low EEG spatial sampling over the cerebellum, and non-realistic spherical head models for source analyses [reviewed in (157)]. However, more and more evidence supports the feasibility of EEG and MEG studies of cerebellar activity, provided that improvements are made to the usual EEG and MEG techniques (157). Todd and colleagues in their series of EEG studies have taken a first step toward these improvements by placing EEG electrodes more posteriorly in order to improve the chances to record cerebellar activity. However, caution should still be taken regarding the results of source localization, as improvements are still needed to adapt the current models which consider the head as a sphere and poorly integrate the cerebellum.

#### Component n15

Several SVS studies report a negative component with an onset latency around 8 ms and a peak latency of 15 ms. The n15 was best recorded under frontal [(107, 113, 148), Figure 3B] or prefrontal electrodes (42, 110). The vestibular origin of the n15 was confirmed by its presence in a patient with profound hearing loss but preserved vestibular function, and its absence in a patient with hypovestibular function but preserved hearing (107). The n15 amplitude increased in the case of superior canal dehiscence, supporting its vestibular origin (148).

The n15 was first thought of pure myogenic origin (107, 148, 158). Indeed, the n15 recorded frontally was similar in size and morphology to responses recorded around the eyes, and it was modulated by changes in gaze direction. In addition, a patient with superior canal dehiscence showed a very large n15 amplitude (up to 11.8 μV) for SVS at 42 dB above vestibular threshold, which is unusual for neurogenic potentials (107). However, left SVS seemed to induce a more asymmetrical n15 with an earlier contralateral onset (107) or larger responses for left compared to right SVS (114), which suggests that there may be a central origin to this component. By contrast, source localization suggested that the frontal n15 may have a cerebellar and cortical origin (108, 113). In particular, a Brain Electrical Source Analysis (BESA) localized the generators in the contralateral cerebellum or the precentral sulcus (108). A Low Resolution Electromagnetic Tomography Analysis (LORETA) localized the n15 generators in the right precuneus and cuneus (113). Altogether, these results indicate that the n15 may represent concomitant vestibular-induced extraocular, cerebellar, and cortical activations around 15 ms. The cerebellar origin of a component better recorded at the frontal level remains however to be confirmed with the development of electrocerebellogram techniques.

#### Component p20

Components with a latency around 20 ms generally follow those detected near 10 or 15 ms with an inversed polarity under the same or closely located electrodes. An n20 component follows the p15 during whole-body rotations (56, 57, 147, 159) or SVS (137, 138). Likewise, in more recent studies, a positive component with a peak latency of 20–21 ms follows the n15 after SVS (107, 108, 113, 114). A p21 component has been reported under frontal electrodes (107, 108) or under posterior occipital electrodes and right temporal electrodes (113). Applying LORETA localized the p21 generators in the right precentral gyrus, with contributions of the right medial and superior temporal gyri (113).

### Conclusion

Electroencephalography and averaging techniques have proven to be effective to study and assess the spatiotemporal dynamics of vestibular information processing within the first milliseconds after stimulation onset. Short-latency responses with a peak latency under 10 ms have been related to activity in the vestibular nerve or vestibular nuclei. This is in accordance with results from early studies in cats and monkeys where the vestibular nerve was directly stimulated and responses recorded in the animal brain (135, 160, 161). Potentials around 2 ms are likely to reflect vestibular nerve response while components with onsets near 2–3 ms and peak latencies observed within 10 ms after the stimulation are attributed to vestibular nuclei activity. Far-field components best recorded at the vertex, such as the n3 and n5, or recorded over parieto-occipital areas, such as the n6, may reflect vestibular information processing along the brainstem, from the lower pons to the rostral pons or midbrain, respectively.

Responses with a peak latency of 10–20 ms may be myogenic, but also reveal a rapid spreading of vestibular information to the cerebellum and cerebral cortex. To our knowledge, only one group documented cerebellar VestEPs in humans (41, 108, 110, 115, 120, 149, 150). Of note, short latency VestEPs were evoked by SVS or IAS at the mastoids, both techniques targeting the otolithic receptors, so that we lack information about the potential cerebellar evoked responses to semicircular canal stimulation. More systematic studies using stimulation techniques such as rotations are needed to better identify the origin of these responses in humans.

Several studies also suggest that vestibular information reaches the cerebral cortex within 6 to 15 ms after stimulation (107, 108, 113, 133). de Waele et al. (133) argued for simultaneous activation of several trisynaptic vestibulo-thalamo-cortical pathways, indicating that vestibular information rapidly spreads to different areas in the cortex. However, the hypothesis of a parallel processing in these areas is not consistent with recent electrophysiological studies in monkeys. Parieto-insular vestibular cortex responses to translations had shorter latency compared to responses in the ventral intraparietal (VIP) area and area MST, supporting the idea that the PIVC is “closer” to the periphery (23).

## Middle Latency Vestibular-Evoked Potentials

Only few studies identified VestEPs with a latency between 20 and 50 ms. Here, we will focus on two biphasic VestEPs that seem to consistently emerge from EEG studies: a first VestEP with peak latencies around 20 and 30 ms and a second VestEP with peak latencies around 42 and 52 ms. Some studies also described individual peaks with similar latencies separately.

### The “20–30 ms Complex”

**Figure 4:**
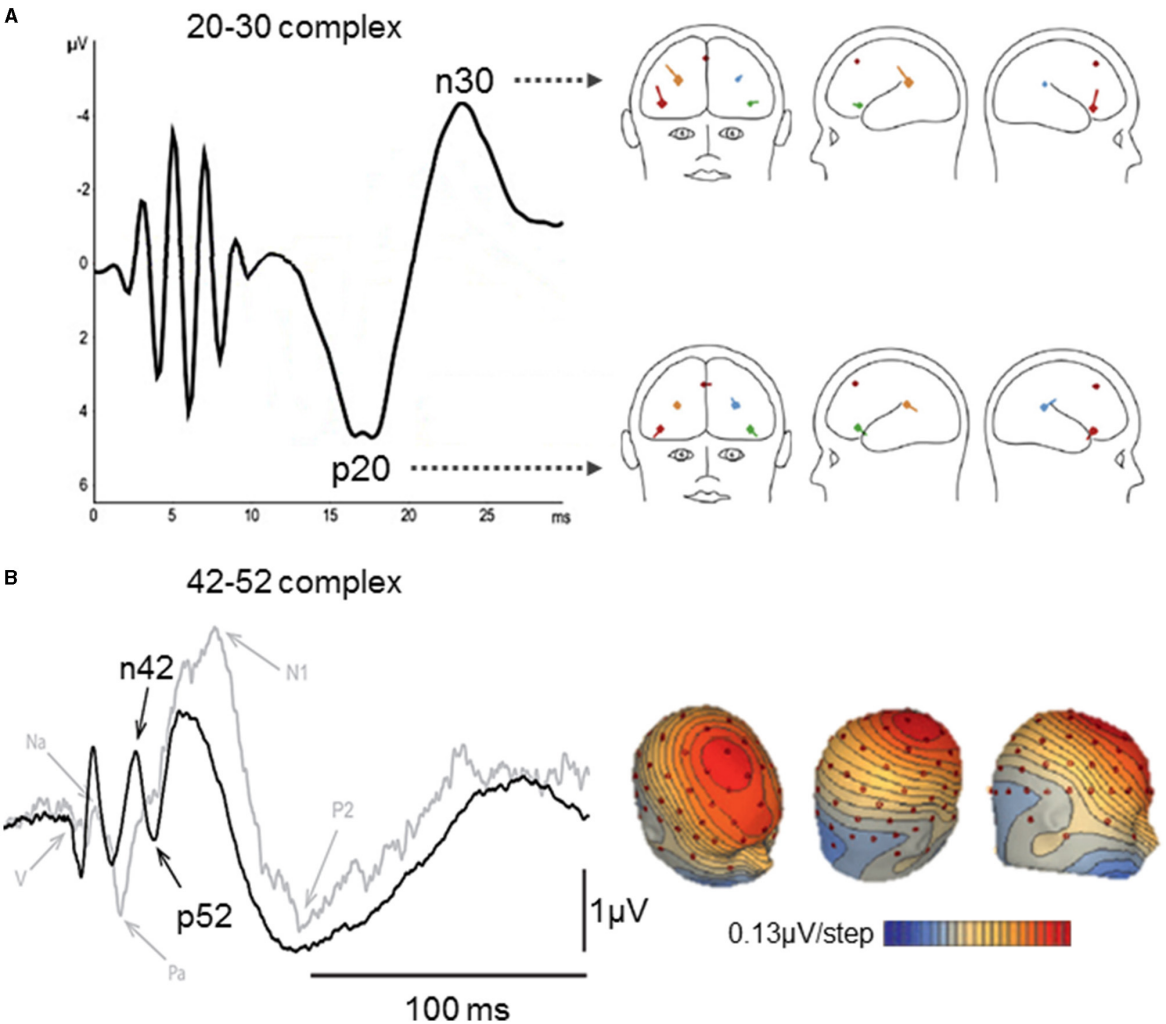
Middle latency vestibular-evoked cerebral potentials (VestEPs). **(A)** The left part shows the 20–30 complex observed with a Laplacian montage (FC1 + FC2 + IO) – (TP9 + TP10), after short latency responses appearing within 10 ms. The right part shows BESA regional source activity for the p20 and n30 components, showing the junction of the anterior insula with the inferior frontal gyrus in the right hemisphere (bright red), the posterior parietal operculum (bright blue), a frontal source near areas 2v, 3aNV, and the frontal eye fields (dark red), the left inferior frontal gyrus (green), the left parietal operculum (brown). Adapted from (111) with permissions from Elsevier. **(B)** Left panel: Grand means of evoked potentials showing the n42–p52 response under electrode FCz after right acoustic stimulation in 10 healthy subjects (black curve) and a patient with a unilateral vestibular loss (gray curve). Right panel: Scalp voltage maps at 42 ms after acoustic stimulation (+18 dB above the vestibular threshold). Positive potentials are indicated in blue and negative in red and contours are spaced at 0.13 μV. Adapted from (42).

A 20–30 ms complex was reported after SVS (111, 162), GVS (96), and skull vibration at the nasion (112). Sound-induced vestibular stimulation evoked a positive peak at 20 ms and a negative peak at 30 ms under fronto-central and centroparietal electrodes of a 32-channel EEG [(111); Figure 4A]. This 20–30 complex was also observed using a simplified Laplacian montage that could be used for clinical purposes (111). Another SVS study reported the p23, n24, and n32 components separately (108). Galvanic vestibular stimulation elicited a counterpart of the 20–30 ms complex with latencies of 25 and 35 ms (96). A BESA source analysis and results from an epileptic patient implanted with deep brain electrodes revealed that this complex originated from the bilateral anterior insula and posterior operculum (111). However, the comparison of source analyses revealed dipoles oriented differently in space in these regions for same latencies after SVS and GVS (96).

Skull vibration induced biphasic n26–p30 or p26–n35 responses for positive and negative stimulation polarity, respectively (112). Source localization revealed deep midline sources plausibly representing activity from the cingulate cortex, medial thalamus or basal ganglia, as well as bilateral frontal sources (112). Similar independent components were identified near 30 ms using whole-body rotations (147) and translations (163).

### The “42–52 ms Complex”

Several recent studies identified a frontocentral n42–p52 complex using SVS (42, 108, 110) and IAS at the nasion (112). The n42–p52 complex was also referred to as the N^*^/P^*^ response as it appears among auditory EPs. However, it was argued that the n42–p52 (N^*^/P^*^) complex represents a more specific vestibular response as its peak-to-peak slope increased linearly for SVS above the vestibular threshold, and it was not observed in a patient with an unilateral vestibular loss stimulated in the damaged ear [(42); Figure 4B]. Brain Electrical Source Analysis revealed that the n42/p52 was best explained by a mid-cingulate source, together with bilateral sources in the superior temporal cortex (42, 110). This is consistent with fMRI studies showing activity in the cingulate cortex following CVS (75, 164, 165) and GVS (89). Other studies reported positive individual components around 40 ms following whole-body rotations [(163); 38.9 ms] and translations [(33, 166, 167); p38.2 ms under parietal electrodes]. More recently, IAS on the left mastoid was also found to evoke n25, p40, and n53 components under Bz, an electrode placed over the cerebellum, two rows below Iz at the midline [(150); following a nomenclature proposed by Heine et al. (168)].

### Conclusion

Four peaks are most consistently reported as VestEPs of middle latencies: they constitute the 20–30 complex and the 42–52 complex. These components presented little variability in their latencies and their amplitude increased with stimulation intensity, leading the authors to propose them as reliable markers of cortical vestibular information processing (112).

Studies of middle latency VestEPs localize generators in the operculo-insular complex and cingulate cortex, two key areas of the vestibular cortical network. The insular and cingulate contributions to vestibular processing is well-supported by anatomical evidence in non-human primates (20), meta-analyses of neuroimaging data (15, 16), and intracranial electrical stimulation in epileptic patients (169, 170).

## Late Latency Vestibular-Evoked Potentials

Responses with a latency above 50 ms were already identified in VestEPs investigations from the 1960s (47, 49, 50, 95, 137, 138). However, these early studies did not report the latency of all observed components or mentioned series of components within a time range. Studies using whole-body rotations identified five to seven waves with latencies ranging from 70 to 850 ms (44, 171–176). Responses with latencies up to 3,000 ms have been reported (47). We summarize below some of the most consistently reported responses with latencies above 50 ms (see Supplementary Table 1).

### Responses at 60–70 ms

**Figure 5:**
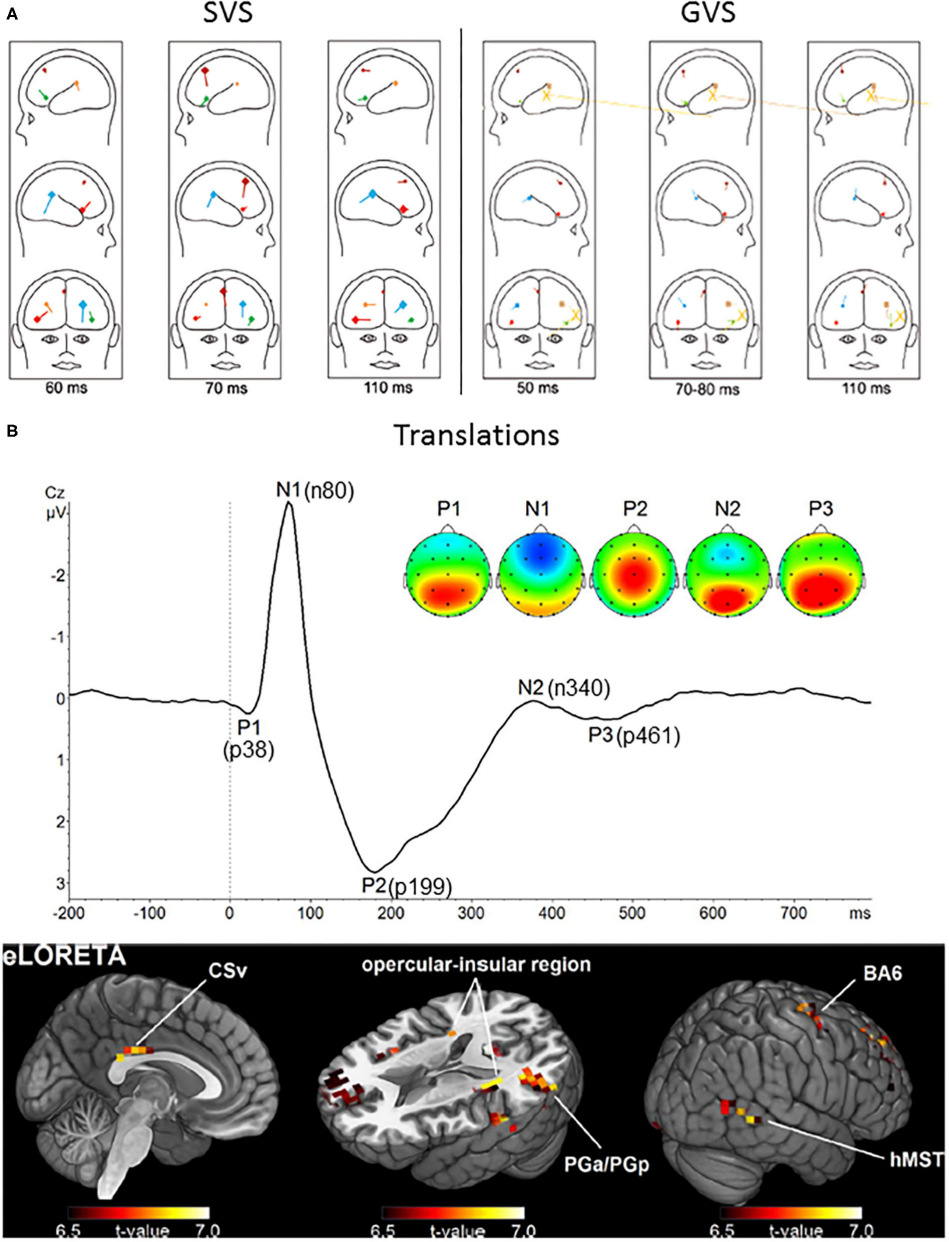
Long latency vestibular-evoked cerebral potentials (VestEPs). **(A)** Sources of evoked potentials observed after SVS (left) and GVS (right). For SVS, BESA regional source activity projected to a head showing the junction of the anterior insula with the inferior frontal gyrus in the right hemisphere (bright red), the posterior parietal operculum (bright blue), a frontal source near areas 2v, 3aNV, and the frontal eye fields (dark red), the left inferior frontal gyrus (green), the left parietal operculum (brown). For GVS: frontal source (dark red), right anterior insula (light red), left anterior insula (light green); posterior operculum (light blue), left posterior operculum (brown). The yellow large dipoles marked with X represent the combined bilateral bipolar capacitive effects of the galvanic pulse removed with principal component analysis. Adapted from (96, 111). **(B)** Upper panel: Grand average response to translation recorded under electrode Cz. Four late latency VestEPs (n80, p199, n340, and p461, originally referred to as N1, P2, N2, and P3) appear after the middle latency component p38. Their characteristic topographies can be distinguished (positive in red, negative in blue). The p38, n340, and p461 components are dominated by strong bilateral activity (red) over parietal regions. The n80 component is best described by a negative potential (blue) detectable at frontal electrodes. The p199 component has a strong positive peak at electrode Cz. Lower panel: The mean activity of all five components suggests that the cingulate sulcus visual area (CSv), the opercular-insular region, Brodmann area (BA) 6, the inferior parietal lobule (PGa/PGp), and the human medial superior temporal area hMST are the main nodes in the processing of otolithic signals. Adapted from (33) with permissions from Academic Press.

Sound-induced vestibular stimulation has been shown to evoke a positive component with a peak latency of 60 ms under temporal electrodes, followed by a frontal component with a peak latency of 70 ms (111). The authors hypothesized that the 70 ms component may reflect crosstalk activity between vestibular areas 2v, 3nv, and the frontal eye fields (111). Galvanic vestibular stimulation also evoked responses at 50 and 80 ms under the same electrodes (96). Source analysis as well as data from an epileptic patient implanted with deep electrodes showed that the anterior insula and posterior opercular cortex responded to SVS [(111); Figure 5A]. Similar sources were observed for the response to GVS [(96); Figure 5A]. Impulsive acceleration stimulation evoked VestEPs with similar latencies under electrode FCz (112). These VestEPs consisted in a negative peak at 65 ms after positive polarity stimulation and a positive peak at 60 ms followed by a negative peak at 78 ms after negative polarity stimulation (112). Source analysis suggested a posterior cingulate contribution.

### Responses at 80 ms

Whole-body translation evoked the n80, a negative component with a peak latency of 80 ms [(33, 166, 167); Figure 5B]. The n80 was most prominent under frontal electrodes, but a weaker occipital positivity was also observed with the same latency. The n80 amplitude increased with linear acceleration intensity (33, 166). This was explained by increased activity in the cingulate sulcus visual area, an area involved in self-motion processing (177).

### Response at 110 ms

A positive component with a peak latency at 110 ms was reported after both SVS and acoustic control stimulation (111), as well as after GVS (96). This suggests a vestibular contribution to the late auditory EPs, around 100 ms.

### Responses at 200 ms

An early study used a vertex referred to mastoid montage and showed a positive potential with a peak latency of 220 ms after the sudden stop of a rotation (178). This VestEPs was present in 30 healthy participants, but it was absent in two patients after labyrinthectomy. A subsequent study compared the p220 in 159 patients with infarct on the middle cerebral artery to those of 130 controls (179). Hundred and twelve patients showed delayed, decreased, or no evoked response at all, suggesting that vestibular evoked responses to rotations involved the temporo-parietal cortex. Hofferberth [(179), p. 125] concluded that there is a “*primary pathway of vestibular evoked potentials […] from the vestibular nuclei via the midbrain to the thalamus and from the thalamus to the temporo-parietal cortex*.” This is very close to some descriptions of vestibular pathways, highlighting a main contribution of the temporo-parietal cortex (65, 89, 180, 181).

More recent studies using body translations revealed a positive component with a peak latency of 199 ms, best observed under electrode Cz [(33, 166, 167); Figure 5B]. As for the n80, the p199 amplitude increased with body acceleration and this increase was best explained by increased activity in the cingulate sulcus visual area.

Finally, we note that other EEG investigations using whole-body rotations have identified independent components with peak latencies at 200 ms (50, 182), or biphasic waves with peak latencies from 200 to 350 or 400 ms, maximally recorded at the vertex (49, 52, 178, 179, 183, 184).

### Responses at 300–500 ms

Various responses have been described within the 300–500 ms time window after active (58, 59) and passive body rotations (32, 44, 47–49, 51, 52, 171, 174, 176, 185), as well as after body translation (33, 166, 167, 186). A seminal study compared human responses to animal responses that were already accepted as vestibular in origin (48). The authors used the sudden stop of yaw rotations as a stimulus both in cats with implanted electrodes and in humans who underwent scalp EEG recordings. They observed late diffuse responses with peak latencies of 300–600 ms in both species, which were prevalent in the preoccipital (area 19) and/or parastriate (area 18) regions in humans. Vestibular-evoked potentials with such late latencies likely reflect the processing of the acceleration profile, with peak amplitudes at peak accelerations (186) or movement inversion (32, 47).

Although their exact latency differ between studies, VestEPs around 300 ms have consistently been reported after passive whole-body rotations (32, 185) and translations (33). For example, whole-body rotations in the yaw plane evoked a vertex negative component with a peak latency near 300 ms (32, 185).

Similarly, whole-body translations evoked a negative component at 340 ms under parietal electrodes, accompanied by a weak negativity under electrode FCz, as well as a positive component at 461 ms which was best observed in centroparietal regions [(33); Figure 5B].

### Conclusion

Late VestEPs are less well characterized than short and middle latency responses and they appear during a large time window after the stimulation onset. Based on two recent studies using body translations, Ertl et al. (166) proposed that responses with a latency under 220 ms may reflect physical properties of the stimulus, whereas later responses may reflect higher level perceptual and cognitive processes. Such transition from a sensory processing to a higher-level perceptual analysis has been proposed for the auditory system (187).

## Conclusions and Perspectives

We have reviewed results from pioneering electrophysiological studies and more recent studies using state-of-the-art EEG indicating that VestEPs can now be considered meaningful electrophysiological signatures of vestibular information processing from the vestibular nerve to the cerebral cortex, owing to the high temporal resolution of EEG. After summarizing the main findings regarding VestEPs with components of short (<20 ms), middle (20–50 ms), and late (> 50 ms) latency, we discuss how VestEPs studies are informative regarding the parallel vs. hierarchical processing of vestibular signals in the cerebral cortex, and regarding hemispheric dominance of vestibular information processing. While underlining issues with replicability and variability of VestEPs across studies, we discuss the main limitations of VestEPs studies and highlight the difficulty to isolate components of vestibular origin while controlling for extravestibular sensory contributions. Finally, we open perspectives regarding the contributions of VestEPs studies to the fields of neurology, otoneurology, cognitive neuroscience, and systems neuroscience.

### Main Findings and Their Link With Vestibular Processing Along Ascending Pathways

Vestibular-evoked potentials with short, middle and late latency mark the different steps and the spatiotemporal organization of vestibular information processing. Vestibular-evoked potentials with a short latency under 20 ms are the most investigated, but independent potentials or biphasic waves also emerge from the literature in the middle and late latency ranges.

Consistent with electrophysiological results from animal studies (152, 153), the first potentials around 2 ms likely reflect vestibular nerve activity, whereas following potentials around 3 ms indicate information processing in the vestibular nuclei (40, 56, 57, 104, 109, 132, 133, 146, 147). It has been proposed that components found at 5 and 6 ms may reflect the transmission of vestibular information along the brainstem (105, 106). Others proposed that vestibular signals reach the cortex as early as 6 ms after stimulation onset (133), that is with a latency equivalent to that necessary to trigger vestibulo-ocular and vestibulocollic reflexes. Reaching the cortex with such short latency may involve vestibular information in an alarm or preattentional system, in addition to top-down control of vestibulo-ocular and vestibulocollic reflexes.

Several components with peak latencies between 10 and 20 ms appear to reflect parallel spreading of vestibular information to the cerebellum (41, 108, 110, 115, 120, 149) and to several cortical regions, including the precentral sulcus, the precuneus, and cuneus, as well as several frontal areas (108, 113, 133). The exact origin and differentiation of these potentials remain to be clarified, and their cerebellar and cortical generators need to be disentangled from myogenic contributions (41, 108, 115). Alternatively, VestEPs with such latency could reflect the descending control of vestibular oculomotor reflexes.

Middle latency VestEPs (20–50 ms) have been associated to activity in the insular, posterior opercular and cingulate cortex in a series of recent EEG studies using SVS and GVS (42, 96, 111). These areas form the core region of the cortical vestibular network (11, 15, 16, 18, 188) and show strong functional connectivity with other cortical vestibular areas (189).

Results concerning VestEPs with late latencies (>50 ms) are more disparate. The heterogeneity of results from early studies may be due to differences in stimulation techniques, stimulation parameters for the same techniques, different levels of quality for control conditions and limitations of the recording systems/montages (placement and number of electrodes). Recent studies have identified more consistently several VestEPs components with late latency, which are informative about the later steps of vestibular information processing. A major finding about VestEPs with late latency is that components under 220 ms may reflect low-level sensory processing while components above 200 ms may reflect higher level perceptual and cognitive processes, and the conscious processing of vestibular information (166).

### Parallel vs. Sequential Vestibular Information Processing in the Cerebral Cortex

One important question in vestibular neuroscience was to determine whether vestibular signals are transmitted to cortical areas through several parallel pathways with similar latencies, or whether vestibular signals reach a core vestibular area (activated earlier) before being distributed to secondary areas (activated later). The ability to answer this question depends on the temporal and spatial resolution of the recording technique.

Results from VestEPs studies reported above indicate that vestibular signals rapidly reach the cerebral cortex. The observation that several cortical areas may receive vestibular information in <10 ms after direct electrical stimulation of the vestibular nerve has been used to support the idea that there is no primary vestibular cortex, but rather parallel processing of vestibular signals in at least five cortical areas (133). This seems consistent with local field potentials recorded with overlapping latencies in several brain areas after electrical stimulation of the vestibular nerve in rats (136).

Our review of the literature shows that short, middle and late VestEPs have been found to originate from various cortical areas at similar or close latencies, such as both frontal and occipital activations at 10 ms, or later at 80 ms, for example. The studies available to date suggest that VestEPs with latencies between 10 and 20 ms may have a cerebellar origin (41, 108, 110, 115, 120, 149, 150), and/or a cerebral origin with several generators in the precentral sulcus (108) or the precuneus and cuneus (113) for the n15. Vestibular-evoked potentials with latencies from 20 to 30 ms have been associated to activity in the bilateral anterior insula and posterior operculum (111) while components with latencies from 40 to 50 ms have been associated with a mid-cingulate source with contributions of the bilateral superior temporal cortex (42, 110). Vestibular-evoked potentials with late latencies have been associated to various sources in all lobes. Altogether, these studies indicate a rapid diffusion of vestibular information in different areas of the cortex, distributed processing, and crosstalk mechanisms with recurrent processing lasting several hundreds of milliseconds after the stimulation.

The surface EEG and the event-related approach may lack the resolution to fully describe the spatiotemporal pattern of vestibular information processing as it reflect activity of too large assemblies of neurons. Local field potentials and single neurons recordings have a more appropriate spatiotemporal resolution. To our knowledge, local field potentials from intracranial electrodes were recorded in only one epileptic patient during SVS, providing similar findings as recordings from scalp electrodes in healthy participants (111). Interestingly, single neuron recordings in non-human primates have been compared in several cortical areas during natural whole-body displacements. A notable study in macaque by Chen et al. (23) compared the spatiotemporal tuning of neurons from the PIVC, area MSTd (dorsal part of area MST), and VIP during animal translations. On the basis of the response latency of the neurons, the authors propose that there is a “hierarchy in cortical vestibular processing, with PIVC being most proximal to the vestibular periphery and MSTd being most distal.” Accordingly, vestibular signals would be first processed in the PIVC before being transmitted to area VIP and MSTd. Although this seems in contradiction with results from most VestEPs studies summarized here, it may just reflect the inability of EEG to grasp different patterns of responses of different cortical areas to acceleration, velocity, and position of whole-body motion as can do single neuron recordings (24).

Finally, we note it is difficult to compare the spatiotemporal dynamics of vestibular information processing as revealed by EEG analyses with data from fMRI and PET studies of the vestibular cortex. Given the low temporal resolution of these neuroimaging techniques, they have not been able to precisely describe the responses of vestibular cortical areas as a function of time, and never with the time resolution of the VestEPs summarized above. A study by Klingner et al. (190) analyzed the temporal pattern of several cortical responses measured with fMRI during 30 s of CVS using independent component analysis. The authors identified seven independent components representing cortical responses with different temporal profiles. Although the time course of these components differed significantly, with a trend for more lasting response for the component originating from the insula, retroinsular cortex, and superior temporal gyrus, there was no difference in the latency of the peak of the response for all components. Another study combining repeated short pulses of CVS and fMRI showed that during the 40 s following CVS there was a longer and stronger activation in the brainstem compared to activation in the cerebellum, thalamus, and right insula (78). The latency of the peak of the response and the response duration (>10 s) in these two studies can in no way be compared with the electrophysiological findings reported in our review of the literature. Altogether, this indicates the complementarity of EEG and fMRI approaches to better characterize the time course and location of vestibular information processing in the human brain.

### Laterality of Vestibular Projections and Hemispheric Dominance in the Vestibulo-Thalamo-Cortical System

Another important question for vestibular neuroscience concerns the laterality of vestibular projections from one labyrinth to the cortex, and whether there is an overall hemispheric dominance for vestibular information processing, as shown by previous neuroimaging and anatomical studies (77, 102, 191).

Vestibular-evoked potentials with latencies under 10 ms can be recorded bilaterally, but larger amplitudes have been observed ipsilaterally to the perioperative stimulation of the vestibular nerve (133). This is in line with the projection of primary vestibular afferents to the ipsilateral vestibular nuclei, and the inhibition of contralateral vestibular nuclei through rich commissural pathways (192).

Studies of VestEPs with latencies between 10 and 20 ms have reported contradictory results as to the lateralization of the responses. Concerning the p10 evoked by SVS, some studies suggested larger ipsilateral responses with a right hemisphere dominance (107), in line with previous neuroimaging findings using the same type of SVS (102). Other studies found a contralateral dominance of a deep source, potentially cerebellar, for the same component (108, 110, 115). Using IAS, Todd et al. (120) reported an ipsilateral p12/n17 and a contralateral p19/n23, but only reported a contralateral p12/n17 in a subsequent study (150). They found that these EPs of potential cerebellar origin were larger on the right side for bone conducted sounds (108), an observation not confirmed with stronger vestibular stimulation (41). Secondary vestibular fibers project bilaterally to the cerebellum in both animals and humans (17, 192, 193). However, unlike in humans, animal studies converge on larger contralateral responses to vestibular stimulation in the vermis and flocculus (194). Neuroimaging studies have also suggested a contralateral activation of the cerebellum (78).

The few studies that have associated short latency VestEPs with cerebral sources seem to corroborate neuroimaging findings of bilateral responses, with larger responses in the ipsilateral and in the non-dominant hemisphere (77, 102). de Waele et al. (133) reported a larger p10 under ipsilateral compared to contralateral frontal electrodes. As expected, BESA revealed bilateral sources, with ipsilateral sources in the superior frontal gyrus and precentral gyrus, and contralateral sources in the anterior supplementary motor area near the frontal eye fields and in the superior occipital gyrus as well as a transverse prefrontal source (133). The n15 and p20 evoked by right ear SVS were found to originate from the right precuneus and cuneus and from the right precentral gyrus, medial and superior temporal gyri, respectively (113).

There are scarce data on the middle latency VestEPs. Regarding the 20–30 complex, larger amplitudes were observed in the anterior insula for left SVS compared to right SVS in right-handed participants (111). However, such lateralization effects were not reproduced with GVS (96) and IAS (112). Regarding the n42/p52 VestEPs, a weak right ear advantage has been reported for the peak-to-peak amplitude measured at electrode FCz, whereas a contralateral left ear advantage has been found for the n42 component when considered alone (110).

Recent studies on late VestEPs report no hemispheric dominance or contradictory results. Contralateral SVS evoked higher VestEPs amplitudes in the non-dominant hemisphere (111) but these results were not reproduced using GVS (96). Ertl et al. (166) found an effect of the direction of lateral passive translation on two components, the n80 and a positive component with a peak between 240 and 352 ms. Both VestEPs showed larger amplitudes under C3 (left to Cz) or C4 (right to Cz) depending on whether the participants were moved to the left or to the right, respectively. The effects were discussed in terms of lateralized somatosensory stimulation during body motion.

Altogether, results from the VestEPs studies indicate that components with different latencies have bilateral brain sources, thus information originating from one ear is processed in both cerebral hemispheres. The analysis of VestEPs amplitudes and their generators, however, is less conclusive regarding the determinants of hemispheric dominance in the vestibulo-thalamo-cortical system, when compared to the clear pattern that arises from PET and fMRI studies: (1) bilateral but larger activations in the hemisphere ipsilateral to the activated ear and (2) larger response in the non-dominant hemisphere, that is in the right cerebral hemisphere for the right-handed participants (77, 195, 196). We agree with Kammermeier et al. (111) in that this difference may in part be due to the “modalities of short-termed electric activity [for EEG studies] vs. long-term vascular or metabolic changes [for fMRI and PET studies].” More work is needed to investigate how VestEPs are influenced by the side of stimulation and the participants' handedness.

### Replicability and Variability of Findings in VestEPs Studies

It may seem that a lack of replicability and large variability of VestEPs emerges from the collection of studies on the topic so far. First, we note that there are few available studies on VestEPs compared to the wealth of studies existing for well-defined visual, somatosensory, and auditory EPs (31). Second, a major obstacle to replicating results from one study to another is the enormous variability in the stimulation and recording procedures. Among the many sources of variability in the recorded vestibular responses, two categories seem particularly important.

#### Method of Vestibular Stimulation

The method of vestibular stimulation (see Figure 1) provides a major source of variation in the observed responses. As noted earlier, artificial stimulation of the vestibular system (GVS, CVS, SVS, IAS, MVS) differ from natural vestibular stimulation (rotatory chair, motion platform) in many aspects, including the type of vestibular receptor (otolithic, canalar) stimulated, the physical nature of the stimulation leading to activation of hair cells or the vestibular nerve, the co-activation of other sensory systems (such as hearing for SVS and interoception for body rotations), the duration of the stimulation, etc. The most commonly used artificial vestibular stimulation for VestEPs studies (SVS and IAS) are transient stimuli lasting in general a couple of milliseconds, whereas passive whole-body translations and rotations have a totally different temporal pattern, lasting in general more than 1 s [e.g., (32, 60)]. It is therefore difficult to directly compare the spatiotemporal dynamics of vestibular information processing for transient artificial vestibular stimulation and natural body motions (see below).

#### The Spatial Density of Recording Electrodes and the Montage

Very diverse electrode montages have been used to record VestEPs with a large variability in the spatial density of electrodes, as shown in Supplementary Table 1. Early studies have used montages close to those used for BAEP recordings, with a spatial density of electrodes insufficient to describe the spatiotemporal dynamics of vestibular information processing. As studies have used different references for the analysis of VestEPs (ear lobes, mastoid, average reference…), it is difficult to directly compare the shape and polarity of VestEPs components across studies. A consensus about standard electrode montage and reference should therefore be established in order to increase replicability of the results in future EEG studies of the vestibular system, as done for cVEMPs and oVEMPs recordings (43, 100), or for somatosensory (34), visual (35), and auditory (36, 37) EPs recordings.

The largest variability in the VestEPs waveforms arises from studies that investigated vestibular responses during natural whole-body translations and rotations. For example, studies of rotatory VestEPs identified only short or middle latency components (56, 57, 147), while others only identified late components (32, 44, 171, 173, 174, 176). This is very likely related to different stimulation parameters. In the case of short and middle VestEPs, the rotations applied to the head only were rapid horizontal (56, 57) or vertical (159) rotational acceleration impulses up to 12,000°/s^2^ that could be applied at frequencies from 0.5 to 2 Hz. In the case of late VestEPs, whole-body rotations could consist in ramps with acceleration phases of 250 or 500 ms to reach an angular velocity of 60°/s maintained for 400 ms (171), step-wise accelerations of 53°/s^2^ (173, 174) lasting 1 s, or accelerations of 15°/s^2^ lasting 1 s (44) or 2 s (176), or transient “raised-cosine” rotations with a peak velocity of 110°/s for 1.3 s (32). Shorter and more intense rotations would therefore make it possible to observe short or middle latency VestEPs while longer rotations would allow to record later responses, possibly time-locked to changes in the acceleration profile.

More consistent results have been obtained with stimulation methods that use standardized stimuli such as short high sound pressure clicks at intensities around 100 dB-SPL. There is indeed a consensus about the parameters of the sounds (frequency, duration, intensity, number of stimulation) which are optimal to activate the otolithic receptors, and are commonly used for clinical investigations of cVEMPs and oVEMPs (38). Vestibular-evoked potentials have been consistently identified using very similar stimulation or recording techniques. For example, the same group has repeatedly reproduced the p10, n15, p21 components as well as the n42/p52 complex using bone conducted or air conducted tone bursts of 500 Hz lasting 6 ms (107, 114) or tone pips of 500 Hz lasting 2 ms (42, 108, 110). Different results were obtained using different parameters of SVS. They compared air-conducted and bone-conducted SVS, showing that they allow to record reproducible components such as the p10 and n15 while noting that bone conducted stimulation induces larger responses (108). They also compared left vs. right SVS (110), different references such as the linked earlobes or common average reference (42), and different inter-stimulus intervals (149). This shows how important the stimulation techniques and their parameters are to better characterize VestEPs.

### Main Limits of VestEPs Studies

The study of the spatiotemporal dynamics of vestibular signal processing in the brain is limited by the fact that the vestibular system is multisensory in nature (197). Vestibular afferents project to the vestibular nuclei and cerebellum, where vestibular signals are processed and integrated with visual and somatosensory information (198). Vestibular signals are also integrated with visual and somatosensory signals in several thalamic nuclei and in several cortical areas, including the PIVC (199). This multisensory convergence can be evidenced by the modulation of the p12/n17 biphasic wave by optic flow (120).

As vestibular signals are mixed with other sensory information as early as the second synapse in the brainstem, most VestEPs recorded to date likely represent a multisensory response owing to the lack of specificity of the methods to stimulate the vestibular receptors (Figure 2). Natural and artificial vestibular stimulation often stimulates one or several extravestibular sensory systems, such as the auditory (for SVS, CVS, IAS, rotating chairs, and motion platforms), tactile (for GVS, CVS, IAS, rotating chairs, and motion platforms), nociceptive (for GVS and CVS), thermoceptive (for GVS and CVS), and interoceptive systems (for body rotations and translations). Thus, vestibular responses recorded over the scalp are mixed with time-locked responses from these sensory systems. During passive whole-body translations and rotations, a major sensory influence on VestEPs comes from the interoceptive system, as the body fluids move in a time-locked manner with the rotatory chair or motion platform. The interoceptive and vestibular systems are largely interconnected at both anatomical and functional levels. Interoceptive signals from visceral receptors can modulate vestibular signals as early as in the vestibular nuclei (200). Vestibular information is also integrated with interoceptive signals in the insula (80). Although visual, auditory, tactile, and nociceptive controls can be used, it is impossible to control for the interoceptive contributions to VestEPs, which cannot be switched off and are hardly manipulated. For example, late VestEPs responses are certainly not purely vestibular as they can also be observed in patients with a bilateral vestibular failure (32, 182).

### Potential Applications of VestEPs in Neurology and Otoneurology

Despite these limitations, VestEPs represent a promising tool for the clinical investigation of the vestibular system. First, there seems to be little interindividual differences in VestEPs onset and peak latencies for electrical stimulations of the vestibular nerve (133) and for SVS (42, 110, 149). Second, VestEPs amplitude increases with the intensity of the electrical current applied to the vestibular nerve (131, 133), the intensity of SVS (42, 107), the impulsive acceleration in IAS (112) or the acceleration of the body rotation (44, 173, 184) or body translation (33, 60). The consistency of these characteristics makes VestEPs measurement appropriate for basic research and clinical investigations. Studies in large populations should first be conducted to establish normative values of latency and amplitude in healthy participants as a function of age, as done for VEMPs [i.e., tests of the otolithic function; (201, 202)] and for the video head impulse test [i.e., tests of the semicircular canals; (203)].

Sound-induced vestibular stimulation appears a convenient and reliable technique to standardize VestEPs as done for cVEMPs and oVEMPs, for which SVS is already routinely used in clinical testing. With many controlled repetitions possible in a relatively short period of time, SVS evokes short, middle, and long latency VestEPs which likely reflect the successive processing of vestibular information along the vestibulo-thalamo-cortical pathways (see Figure 1). Sound-induced vestibular stimulation allows to test each ear separately. Simplified montages could be used, such as Laplacian montages (111), or specific vertex-mastoid or parieto-frontal derivations for example (106). Interestingly, recordings with 32- and 64-channel EEG systems allowed to observe a reduction of the n42/p54 response in few patients with a bilateral (114) or unilateral (42) vestibular loss. A first step toward clinical applications of VestEPs would be to use SVS (and the appropriate auditory control conditions) to test larger samples of patients with various vestibular disorders and compare their responses to those from healthy participants.

Brainstem auditory evoked potentials have been proposed as a complement to cVEMPs to evaluate vestibular schwannoma (204), even though their cost-effectiveness may be lower than that of MRI (205). Compared to cVEMPs, far-field vestibular potentials have the advantage to be recorded even in patients who cannot properly contract their neck muscles, a condition for cVEMPs recording. It has been proposed that the n5 may be suitable to assess vestibular projections in clinical practice. First, the n3 component may only appear when BAEPs auditory components are drastically reduced. Second, the n5 can be observed with sounds of lower intensity, around 80 dB nHL, and allows for shorter testing times compared to the n3, which is best obtained using clicks over 100 dB nHL (105).

To record middle and late latency VestEPs in clinical practice, Kammermeier et al. (111) have proposed a simplified EEG setup with circular Laplacian montages around electrodes FC5/6 (over right and left anterior insula) and CP5/6 (right and left posterior opercula). They found reproducible n20, p30, and p60 responses, indicating that VestEPs of short, middle, and late latency can be recorded using simplified montages in clinical settings.

Vestibular-evoked potentials should be altered in patients with lesions in vestibular areas, providing a faster, simpler and less expensive equivalent to fMRI or PET for demonstrating reduced or altered activity in vestibular areas (206, 207). Vestibular-evoked potentials could help assess central vestibular processing in patients who report vestibular sensations but show no apparent vestibular end organs or nerve alterations and help identify the underlying pathology. Vestibular-evoked potentials could be an important diagnostic support in the investigation of central vestibular syndromes (21). Among these, the incidence of vestibular epilepsy is probably underestimated and pose important differential diagnostic problems with vestibular migraine and psychogenic forms of paroxysmal vertiginous manifestations (208, 209). It is possible that, as in other epilepsies, specific alterations of certain cerebral areas may be investigated by VestEPs in the future and that these alterations will be different in migraine and psychogenic disorders. One the one hand, there is evidence that patients with vestibular migraine have a different vestibular threshold and sensitivity to motion than healthy controls (210, 211). On the other hand, there is evidence that patients with persistent postural-perceptual dizziness (PPPD), a functional vestibular disorder (212) showed altered activity and connectivity in the vestibular cortical network, including areas processing visuo-vestibular integration and emotions (213–215). In an fMRI study, SVS evoked reduced activation and connectivity of key vestibular areas such as the posterior and anterior insula, hippocampus and anterior cingulate cortex in chronic subjective dizziness compared to healthy controls (213). We propose that VestEPs may provide more information on the temporal characteristics of vestibular processing linked with anxiety in this form of functional vestibular disorder.

### Perspective of VestEPs for Cognitive and Systems Neuroscience

We propose that VestEPs analysis is a method to investigate the spatiotemporal characteristics of multisensory mechanisms underlying various pre-reflexive and cognitive functions. To meet such objectives, studies need to combine vestibular stimulation with cognitive tasks, as it has already been successfully done. A self-motion oddball detection task combined with VestEPs recording revealed a vestibular-evoked P3 component, a marker of infrequent change detection identified for other sensory modalities (216, 217). A perceptual decision making study (166) showed a positive component with latencies ranging from 240 to 352 ms, that may reflect expectation and decision-making processes involving self-motion signals (166), as it was not observed in previous recordings in participants not engaged in any task (33). Studies of the modulation of known EPs during vestibular stimulation, as well studies of the variations of VestEPs according to different multisensory stimuli or cognitive tasks are therefore feasible and could yield important insights into the vestibular contribution to perception and cognition.

Vestibular-evoked potentials may also offer the possibility to study more precisely the spatiotemporal dynamics of attentional and cognitive effects reported using other neuroimaging techniques. An fMRI study showed that attentional load in a visual tracking task decreased activity in the PIVC (218), possibly due to a downregulation of excitatory neurotransmitters and maintenance of inhibitory transmitters to reduce PIVC responses to thalamic inputs (219). Vestibular-evoked potentials may help to refine the timing of such phenomena as well as to study them in several areas simultaneously.

Although it is beyond the scope of the present review article to describe event-related synchronizations and desynchronizations during vestibular stimulation, response analysis in the time-frequency domain could also serve the same objectives as VestEPs analysis and provide additional information about the spatiotemporal dynamics of vestibular information processing in healthy participants and in patients. Indeed, EPs and event-related synchronizations/desynchronizations reflect different electrophysiological events. Evoked potentials are phase-locked events originating from post-synaptic responses of cortical neurons (220). By contrast, event-related synchronizations/desynchronizations are time-locked events, but not phase-locked events, reflecting more neuronal and synaptic characteristics, network connectivity, and modulation on more or less extended neuronal assemblies (220). A few studies investigated both VestEPs and responses in the time-frequency domain (32, 111, 166, 167, 171). Rotations were found to induce alpha rhythm desynchronization in central and parietal scalp regions (32, 171). This desynchronization was significantly reduced in patients with a bilateral vestibular failure when compared to healthy participants, indicating it is in part related to vestibular information processing (32). A more recent study found that body translations induced a delta and theta synchronization in the bilateral operculo-insular region, mid-orbital gyrus, and medial frontal gyrus, with additional contribution to the theta synchronization from cingulate sulcus visual area and anterior cingulate gyrus (167). Body translations and SVS were both associated with low beta synchronizations, observed at Cz 67.5 ms after maximum acceleration for translations (33), and localized in the right anterior insula and posterior operculum 20–80 ms after SVS (111). Delta, theta, mu, or gamma synchronizations and desynchronizations were also reported following passive body rotations (32, 171), translations (166, 167), and SVS (111). Here, again studies in larger populations, using standardized stimulation and recording techniques, or time-frequency or microstates (166) analyses would be needed to complement VestEPs findings.

Finally, MEG could also be used to study VestEPs and vestibular-related synchronizations and desynchronizations. Magnetoencephalography offers better source localization than EEG and would therefore be a good complement to the spatial localization of vestibular information processing, especially for investigations of deep sources such as the cerebellum (157). However, EEG remains the only recording technique fully compatible with more ecologically valid vestibular stimulation.

### Conclusion

Vestibular-evoked potentials of short, middle, and late latency reveal the spatiotemporal properties of vestibular processing from the vestibular nerve to cortical areas. They represent promising tools for the clinical and experimental investigation of the vestibular system, its disorders and its relation to cognition.

## Author Contributions

EN and CL wrote the draft and revised the final manuscript. FB revised the final manuscript. All authors contributed to the article and approved the submitted version.

## Funding

This work was supported by the ANR VESTISELF project, grant ANR-19-CE37-0027 of the French Agence Nationale de la Recherche to CL and FB.

## Conflict of Interest

The authors declare that the research was conducted in the absence of any commercial or financial relationships that could be construed as a potential conflict of interest.

## Publisher's Note

All claims expressed in this article are solely those of the authors and do not necessarily represent those of their affiliated organizations, or those of the publisher, the editors and the reviewers. Any product that may be evaluated in this article, or claim that may be made by its manufacturer, is not guaranteed or endorsed by the publisher.
