# Supplementary material for: Vestibular-Evoked Cerebral Potentials
Source: Front Neurol. 2021 Sep 21;12:674100. doi: 10.3389/fneur.2021.674100 (PMC8490637; doi:10.3389/fneur.2021.674100)
Supplement: Supplementary file 1 [file Table_1.pdf]

Latency	Stimulation	Study	Component polarity and latency (in ms)*	Component location on the scalp	Electrode montage
Short (< 20 ms)	Intraoperative vestibular nerve stimulation	Häusler et al., 1992	n2	Frontal	Forehead and ipsilateral earlobe, ref.: shoulder
		de Waele et al., 2001	onset: 3–5; peak: 9.5<br><br>onset: 6–7; peak: 10.7	Temporal<br><br>Frontal	30 active electrodes 10-20 system, ref.: nose
	Rotations	Elidan et al., 1991a, 1991b	p3.5, n6, p8.4, p8.8, n18.8, p25.8	Frontal	Forehead-ipsilateral mastoid, ground: contralateral mastoid (pediatric ECG electrodes)
		Rodionov et al., 1996a	onset: 2.2<br>p2.9, p5.1, p7.0, p8.6, p14.5, n19	Frontal	3 disposable pediatric ECG electrodes: 1 on each mastoid, 1 on forehead.
		Rodionov et al., 1996b	p15, n20	Frontal	Disposable pediatric ECG electrodes: 1 on left mastoid, 1 on forehead, ground: right mastoid + EOG
	Translations	Knox et al., 1993	n3 and n6, possible component at 9-10	Frontal	1 test ear, 1 nontest ear, 1 on forehead
	SVS	Kato et al., 1998	n3	Vertex	1 electrode at vertex, 1 at mastoid ipsilateral to stimulated ear, ref.: forehead or contralateral mastoid or C7 or upper margin of the sternum.<br><br>EEG 15 active electrodes (10-20 system), ref.: C7
		Papathanasiou et al., 2003	n3	Parietal	19 Ag/AgCl electrodes (10-20 system), ref.: right hand dorsum
		Papathanasiou et al., 2004	n4.72	Parietal	Ag/AgCl electrodes: P3, P4, ref.: FPz, ground: Cz

	Murofushi et al., 2005			Ipsilateral mastoid and vertex. Ground: nasion.
	Papathanasiou et al., 2010	n5<br>n6	Parieto-frontal derivation<br>Parieto-occipital derivation	Multiple derivations using P3, F4, C4, P4, O2, F8, T4, T6, A2, O1, Fpz, T5, Pz, A1, T3, C3, Cz, F7, F3, Fz (10-20 system), ref.: Fz
	Bickford et al., 1964	onset: 6–8<br>peak: n12, n26	Occipital (inion)	Recording between inion or vertex and left ear
	Cody et al., 1964	onset: 6–11<br>peak: n10-15<br><br>onset: 23-28<br>peak: n25-31	Occipital (inion)	Recording at inion, ref.: earlobe or nose.
	Todd et al., 2003	onset: 7,<br>peak: p10<br>n15<br>n17<br>p21	Vertex (Cz)<br>Frontal (Fpz)<br>Centro-frontral<br>Frontal	9 Ag/AgCl electrodes at Fpz, F3, F4; F7, F8, Cz, T3, T4, ref.: linked earlobes, ground: sternum or inner forearm
	Rosengren and Colebatch, 2006	p10<br>n15<br>p21<br>p31<br>n41	Fronto-central (maximal at Cz)<br>Frontal<br>Frontal<br>Fronto-central<br>Fronto-central	9 Ag/AgCl electrodes at Fpz, F3, F4; F7, F8, Cz, T3, T4, ref.: C7, contralateral earlobe, linked earlobes or sternum, ground: lateral sternum
	Todd et al., 2008	p10-n17<br>n15-p21	Parietal (Pz)<br>Frontal (Fpz)	21 Ag/AgCl electrodes (placed according 10-20 system), ref.: C7
	McNerney et al., 2011	n14.7<br>p 20.7	Frontal<br>Occipital and temporal	64-channel EEG, ref.: common average
	Todd et al., 2014b, 2014c	p10<br>n15	Parietal<br>Prefrontal	64-channel EEG with additional electrodes: 2 infra-ocular, 2 at deep frontal locations and 2 at earlobes, ref.: linked earlobe or average
	Todd et al., 2016a	p10/n17	Occipital	64-channel EEG with additional electrodes: 2 infra-ocular, 2 at deep frontal locations, 2 at earlobes and 2 over splenius muscles of the neck

		Govender et al., 2020	p10/n15	Occipital/Cerebellar	13 Ag/AgCl on each side: Oz, Iz (10-20 system), CBz (midline, 5% of nasion-inion distance below Iz), 3 rows of lateral electrodes with interval ~3cm from midline, 1 over splenius, ref.: ipsilateral earlobe, ground: suprasternal notch.
	IAS	Pyykkö et al., 1995	n1.9, p2.4, n4.5		Vertex-mastoid recording + EOG
		Todd et al., 2018b	p12/n17<br>p19/n23	Occipital/Cerebellar	6 Ag/AgCl: C3/4 (10-10 sytem), CB1/2 (same longitude as PO7/8 on the inferior nuchal line of the posterior fossa), SP1/2 (over splenius muscles) + EOG
		Govender et al., 2020	positive polarity stimulation: p12/n17<br>negative polarity stimulation: p20/n26	Occipital/Cerebellar midline	13 Ag/AgCl on each side: Oz, Iz (10-20 system), CBz (midline, 5% of nasion-inion distance below Iz), 3 rows of lateral electrodes with interval ~3cm from midline, 1 over splenius, ref.: ipsilateral earlobe, ground: suprasternal notch
		Todd et al., 2021	p12/n17	PO10 (cerebellar)	63 channels EEG/ECeG with 10% cerebellar extended 10-10 system
Middle (20-50 ms)	Rotations	Rodionov et al., 1996a	p31	Fontal (forehead)	3 disposable pediatric ECG electrodes: 1 on each mastoid and 1 on forehead
		Trinus, 1997	p38.9		Vertex-mastoid recording
	Translations	Trinus, 1997	onset: 6<br>p30	Vertex	Vertex-mastoid recording
		Ertl et al., 2017	p38.2	Parietal	32 channel EEG (10-20 system), online ref.: FCz, offline ref.: common average
	SVS	Bickford et al., 1964	n54	Occipital (inion)	Recording between inion or vertex and left ear
		Todd et al., 2008	p23, n24, n32, n42, p50	Fpz	21 Ag/AgCl electrodes (placed according 10-20 system), ref.: C7
		Todd et al., 2014b,c, 2016b	n42/p52	Fronto-central	64-channel EEG with additional electrodes: 2 infra-ocular, 2 at deep frontal locations and 2 at earlobes, ref.: linked earlobe or common average

		Kammermeier et al., 2015	n20, p30		32-channel EEG<br>8-channel Laplacian montage<br>+ intracranial recordings from 1 patient
	IAS	Todd et al., 2014a	positive polarity stimulation:<br>peaks: n26, p30, n40, p55<br>negative polarity stimulation:<br>peaks: p26, n35, p40, n50	FCz	64-channel EEG with additional electrodes: 2 infra-ocular, 2 at deep frontal locations and 2 at earlobes, ref.: linked earlobes
		Todd et al., 2021	n25, p40, n53	Bz (nomenclature from Heine et al., 2020)	63 channels EEG/ECeG with 10% cerebellar extended 10-10 system, ref.: Nz, ground: AFz (nomenclature from Heine et al., 2020)
	GVS	Kammermeier et al., 2017	25, 35		32 Ag/AgCl electrodes 10-20 EEG, including right-sided infraorbital EOG channel
Long<br>(> 50 ms)	Passive rotations	Greiner et al., 1967	500 to 1000 after movement inversion or maximal acceleration	Temporo-occipital derivation	5 Ag electrodes for bipolar derivations: 1 at vertex, 2 on each side in the inferior temporal region (2 cm over external auditory canal), 2 in occipital region (4 cm of median line and 4 cm above inion)
		Spiegel et al., 1968b	300–600 ms	Occipital	Several EEG electrodes with at least 1 frontal, 1 temporal and 1 occipital. Ref: chin.
		Salamy et al., 1975	n193, p345		Parasagittal electrodes in 6 Ss (F3, F4, C3, C4, P3, P4), mid-sagittal in 4 Ss (Fz, Cz, Pz), coronal in 10 Ss (T3, C3, Cz, C4, T4), ref.: linked ears + EOG
		Gerull et al., 1981	n200	Vertex	6 different electrode placements from frontal to occipital and neck, ref.: mastoid
		Hood, 1983	onset: 150<br>p500-600	Vertex	Vertex-mastoid
		Hofferberth, 1984, 1995	p220, n300	Vertex	Vertex-mastoid

Hood and Kayan, 1985	n249, p404.6	Vertex	1 electrode at vertex, ref.: nose, ground: nasion + explored other scalp locations (10-20 system)
Pirodda et al., 1987	Different waves from 40 to 350 ms	Vertex	1 electrode at vertex, ref.: ear lobes, ground: forehead
Keck, 1990	200	Vertex	Vertex-mastoid (both sides)
Bertora and Bergmann, 1995	n88.96, n213.21, p289.90, n351.70, n410.15	Central and parietal	8 electrodes at F1, F3, C3, C4, P3, P4, O1, O2 (10-20 system), ref.: mastoids
Kolchev, 1995	77.0, 182.2, 336.6<br><br>476.2, 631.5, 803.0	Central transversal line (T3-C3-CZ-C4-T4)<br><br>Frontal (Fp1, Fp2, Fz)	19 Ag/AgCl electrodes (10-20 system), ref.: mastoids
Claussen et al., 1995	6 to 7 negative/positive waves between 65 and 800 ms	Most prominent in central electrodes	19 electrodes (10-20 system), ref.: mastoids
Trinus, 1997	n81.7, p148.2	Vertex	Vertex-mastoid
Schneider et al., 1996	n77, n182, n336, p475, n631, n802	Central – transversal line (T3-C3-Cz-C4-T4)<br>Frontal	19 Ag/AgCl electrodes (10-20 system), ref.: mastoids
Schneider et al., 2001	n75, n180, n330, p480, n630, n800	Central – transversal line (T3-C3-Cz-C4-T4)	19 Ag/AgCl electrodes (10-20 system), ref.: line connecting both mastoid to rear center of the skull
Kenmochi et al., 2003	onset: 250<br>n293 (triphasic negative-positive waves between 200 and 362 ms)	Vertex	Ag/AgCl electrodes: 1 to vertex, ref.: right mastoid, ground: forehead
Probst et al., 1995	onset: 57.5<br>n127.4	Vertex	Vertex-nasion, ground: Fz

	Probst et al., 1997	n1800	Temporal	21 electrodes located 3cm from each other above central and parietal regions, ground: right earlobe
	Loose et al., 2002	1g: n835 (roll-up); n843 (roll-down)<br>0g: n596 (roll-up);<br>N641 (roll-down)	Vertex	3 electrodes, Cz and 3cm to left and right of Cz, ref.: Fz, ground: between Fz and Cz
	Gale et al., 2015	CW: n100-p184, n301<br>CCW: n47-p166, n303	Vertex	192 channel EEG, online ref.: active ref. electrode pair (CMS-DRL) at apex of skull, offline ref.: average ref. + EOG
Active rotations (head only)	Zangemeister et al., 1986	n85 (very fast movements)<br><br>onset: 123.5<br>peaks: n190, p370	Fronto-precentral and parietal<br><br>Centro-parietal	3 to 5 electrodes in sagittal and transverse positions, ref.: bimastoid common ref. (Cb1 and Cb2) + EOG
	Zangemeister and Hansen, 1990	n66, n178, p295 n424	Parietal	3 to 5 electrodes, ref.: bimastoid common ref (Cb1 and Cb2) + EOG
Translations	Kast and Lankford, 1986	n64-97, p156-190	Temporal	2 active electrodes on each mastoid, ref.: vertex, ground: forehead
	Baudonnière et al., 1999	n86	Vertex (Cz) and frontal (Fz)	21 cutaneous electrodes (10-20 system) ref.: linked earlobe + EOG
	Trinus, 1997	n70.3, p145.5	Vertex	Vertex-mastoid
	Nolan et al., 2011	500, 1000		128 channel EEG + 7 electrodes for EOG, ref.: average
	Nolan et al., 2012	onset: 500<br>peak: 600	Parieto-central	128 channel EEG, ref.: common average + EOG + EMG
	Ertl et al., 2017	n80<br><br>p199<br>n340	Frontal (and weaker occipital positivity)<br>Vertex<br>Parietal (weak at FCz)	32 channel EEG (10-20 system), online ref.: FCz, offline ref.: common average

		p461	Centro-parietal	
	Ertl et al., 2020	n80<br><br>p199<br>p176-240<br><br>p240-352	Frontal (and weaker occipital positivity)<br>Vertex<br>vertex<br><br>Parietal (Pz)	32 active electrodes EEG, ref.: common average
SVS	Bickford et al., 1964	n75 and components < 200 ms	Occipital (inion)	Recording between inion or vertex and left ear
	Cody et al., 1964	onset: 55-95<br>p 125-195	Vertex	Recording at vertex, ref.: earlobe or nose
	Kammermeier et al., 2015	p60<br>n70<br><br>p110	 <br><br>Parieto-temporal	32 active Ag/AgCl electrode EEG + EOG + EMG<br><br>+ intracranial recordings from 1 patient
IAS	Todd et al., 2014a	positive polarity stimulation: peaks: n65<br>negative polarity stimulation: peaks: p60, n78	Frontal (FCz)	64-channel EEG with additional electrodes: 2 infra-ocular, 2 at deep frontal locations and 2 at earlobes, ref.: linked earlobes
	Todd et al., 2021	n107, p178, n293	Frontal (FCz)	63 channels EEG/ECeG with 10% cerebellar extended 10-10 system, ref.: Nz, ground: AFz (nomenclature from Heine et al., 2020)
GVS	Molinari & Mingrino, 1974	onset: 60-80<br>series of positive-negative waves up to 400–500 ms	Median fronto-occipital line	3 needle electrodes along a median fronto-occipital line (2 recording electrodes, 1 frontal and 1 occipital, and 1 guard electrode at vertex)
	Kammermeier et al., 2017	60<br>70, 110	Frontal	32 Ag/AgCl electrodes 10-20 EEG, including right-sided infraorbital EOG channel

**Supplementary table 1. Main components of vestibular-evoked potentials sorted according to their short, middle and late latency for different methods of vestibular stimulation. ***As the labels (P1, N1, P2, N2, etc.) referred to very different latencies in different studies using different paradigms and stimulation parameters, we homogenize the report of the components polarity and latency by indicating positive (p) and negative (n) components followed by their reported peak latency (or average latency) expressed in ms post-stimulation onset. According to our convention, a n80 indicates a negative component with a peak latency of 80 ms and a p199 indicates a positive component with a latency of 199 ms. CW: clockwise; CCW: counterclockwise; C7: seventh cervical vertebra; ECeG: electrocerebellogram; ECG: electrocardiogram; GVS: galvanic vestibular stimulation; IAS: impulsive acceleration stimulation; ref: reference; Ss: subjects; SVS: sound-induced vestibular stimulation.
